# Microstructure—Thermal Property Relationships of Poly (Ethylene Glycol-*b*-Caprolactone) Copolymers and Their Micelles

**DOI:** 10.3390/polym14204365

**Published:** 2022-10-16

**Authors:** Khandokar Sadique Faisal, Andrew J. Clulow, Stephanie V. MacWilliams, Todd A. Gillam, Ashlyn Austin, Marta Krasowska, Anton Blencowe

**Affiliations:** 1 Applied Chemistry and Translational Biomaterials (ACTB) Group, Centre for Pharmaceutical Innovation (CPI), UniSA Clinical and Health Sciences, University of South Australia, Adelaide, SA 5000, Australia; 2 BioSAXS Beamline, Australian Synchrotron, Australian Nuclear Science and Technology Organisation (ANSTO), Clayton, VIC 3168, Australia; 3 Drug Delivery, Disposition & Dynamics, Monash Institute of Pharmaceutical Sciences, Parkville, VIC 3052, Australia; 4 Surface Interactions and Soft Matter (SISM) Group, Future Industries Institute, UniSA STEM, University of South Australia, Mawson Lakes, SA 5095, Australia

**Keywords:** micelle, copolymer, microstructure, thermal behaviour, crystallinity

## Abstract

The crystallinity of polymers strongly affects their properties. For block copolymers, whereby two crystallisable blocks are covalently tethered to one another, the molecular weight of the individual blocks and their relative weight fraction are important structural parameters that control their crystallisation. In the case of block copolymer micelles, these parameters can influence the crystallinity of the core, which has implications for drug encapsulation and release. Therefore, in this study, we aimed to determine how the microstructure of poly(ethylene glycol-*b*-caprolactone) (PEG-*b-*PCL) copolymers contributes to the crystallinity of their hydrophobic PCL micelle cores. Using a library of PEG-*b*-PCL copolymers with PEG number-average molecular weight (*M_n_*) values of 2, 5, and 10 kDa and weight fractions of PCL (*f_PCL_*) ranging from 0.11 to 0.67, the thermal behaviour and morphology were studied in blends, bulk, and micelles using differential scanning calorimetry (DSC), wide-angle X-ray diffraction (WXRD), and Synchrotron wide-angle X-ray scattering (WAXS). Compared to PEG and PCL homopolymers, the block copolymers displayed reduced crystallinity in the bulk phase and the individual blocks had a large influence on the crystallisation of one another. The *f_PCL_* was determined to be the dominant contributor to the extent and order of crystallisation of the two blocks. When *f_PCL_* < 0.35, the initial crystallisation of PEG led to an amorphous PCL phase. At *f_PCL_* values between 0.35 and 0.65, PEG crystallisation was followed by PCL crystallisation, whereas this behaviour was reversed when *f_PCL_* > 0.65. For lyophilised PEG-*b*-PCL micelles, the crystallinity of the core increased with increasing *f_PCL_*, although the core was predominately amorphous for micelles with *f_PCL_* < 0.35. These findings contribute to understanding the relationships between copolymer microstructure and micelle core crystallinity that are important for the design and performance of micellar drug delivery systems, and the broader application of polymer micelles.

## 1. Introduction

Polymeric micelles have been widely studied as drug-delivery platforms as they can provide enhanced drug solubility and bioavailability, can extend circulation times in vivo, can mitigate dosage-dependent toxicity, and can deliver therapeutic payloads with site selectivity when decorated with appropriate targeting ligands [1,2,3]. The thermal properties of block copolymers, specifically their melting and crystallisation behaviour, have been shown to greatly impact the micelle assembly process [4,5,6,7,8], imparting changes in size, morphology, dispersity, and stability [9,10,11,12]. These thermal attributes are dependent on the microstructural characteristics of the block copolymer, such as composition, molecular weight, and the relative weight fraction of the core-forming hydrophobic block [13,14]. In particular, the thermal behaviour of PEG-*b*-PCL block copolymers has been an important area of study given their relevance to micelle drug-delivery systems [15,16,17,18,19,20].

Glavas et al. studied the core crystallinity of a range of polyester-based micelles, including PEG-*b*-PCL and PEG-*b*-ε-decalactone (PEG-*b*-PDL) copolymers with a fixed PEG *M_n_* of 2 kDa [14]. PEG-*b*-PCL micelles with a *f_PCL_* of 0.33–0.67 presented with semi-crystalline cores and the enthalpy of melting (∆H_m_) was found to decrease with increasing *f_PCL_*. In comparison, PEG-*b*-PDL copolymers had amorphous cores. Interestingly, the critical micellisation concentration was typically higher for the PEG-*b*-PDL micelles and the micelle size was found to increase with the weight fraction of the PDL block, which was opposite to the PEG-*b*-PCL copolymers. As a result, the amorphous core PEG-*b*-PDL micelles were found to possess almost twice the loading capacity of the PEG-*b*-PCL micelles and released an aniline pentamer payload faster [4], indicating the potential for increased drug loading and release with amorphous core micelles. Similarly, Glover et al. reported that the core of PEG-*b*-PCL copolymer micelles with a PEG *M_n_* of 2 kDa decreased in crystallinity with increasing *f_PCL_*, with melting endotherms between 40 and 45 °C [21]. Heating of pyrene-loaded PEG-*b*-PCL micelles above this temperature to induce a core switch from crystalline to amorphous resulted in the rapid release of the encapsulated pyrene.

The relationship between polymer microstructure and the melting and crystallisation behaviour of PEG*-b*-PCL polymers has been studied extensively in the bulk phase and compared to PEG and PCL homopolymers and their blends [22,23,24,25,26]. Comparison of the thermal properties of block copolymers with their homopolymer counterparts can assist in the elucidation of the effects of individual block components within a copolymer system. In blends, PEG and PCL homopolymers form highly crystalline and separated microdomains [22,25,26]. In comparison, PEG-*b*-PCL copolymers form imperfect crystal structures due to discrepancies in the order in which the two block components crystallise, resulting in a reduction in the melting temperature (*T_m_*) and ∆H_m_ [17,23,25].

The melting and crystallisation behaviour of PEG-*b*-PCL copolymers have been reported to be predominantly influenced by the molecular weight of the PCL component, although many studies have only focused on a small series of copolymers [13,23,24]. Therefore, there is a need for a systematic investigation of a broad series of PEG-*b*-PCL copolymers, covering a range of hydrophobic weight fractions, combined with different PEG molecular weights. Additionally, few studies have determined the crystallinity of PEG-*b*-PCL copolymers in an assembled micelle state [4,14,21], particularly for a large family of copolymers.

Detailed investigations and comparisons of the thermal and morphological attributes of PEG and PCL homopolymer blends, PEG-*b*-PCL copolymers in the bulk phase, and PEG-*b*-PCL within lyophilised assembled micelles are crucial in establishing a thorough understanding of the structure–property relationships of PEG-*b*-PCL polymers and their micelles. Herein, the thermal attributes of a library of PEG and PCL homopolymers, PEG-*b*-PCL copolymers, and micelles have been determined via DSC, WXRD, and WAXS analysis. The melting and crystallisation temperatures of each polymer, and individual blocks where possible, as well as their associated enthalpy changes, are reported. Furthermore, the order of block crystallisation is reported for the copolymers. Valuable insight into one of the key determinants of micelle formation for the PEG-*b-*PCL copolymer system is provided, which may greatly support the future development of this micelle drug-delivery platform.

## 2. Experimental

### 2.1. Materials

Stannous octoate (Sn(Oct)_2_; 92.5–100%), anhydrous toluene (99.8%), deuterated chloroform (CDCl_3_; 99.8% D), lithium chloride (LiCl; 99%), ε-caprolactone (ε-CL; 97%), benzyl alcohol (99–100.5%), tin(II) 2-ethylhexanoate (92.5–100%), PCL (*M_n_* = 2 kDa), and α-methoxy-ω-hydroxy PEG (*M_n_* = 2 and 5 kDa) were purchased from Sigma-Aldrich (St. Louis, MO, USA). α-Methoxy-ω-hydroxy PEG (*M_n_* = 10 kDa) was purchased from Creative PEG Works (Durham, NC, USA). Ultrahigh purity argon (99.999%) and nitrogen (99.999%) were purchased from BOC (Torrensville, SA, Australia). Poly (methyl methacrylate) (PMMA) standards were purchased from Agilent (Santa Clara, CA, USA). PEG-*b*-PCL copolymers were prepared via ring-opening polymerisation, as previously described [27]. Analytical grade diethyl ether, chloroform, toluene, ethyl acetate, *N*,*N*-dimethylformamide (DMF), acetone, and hexane were purchased from Chem-Supply (Gillman, SA, Australia. All reagents were used as received unless otherwise stated. Ultrapure water with a resistivity of ≥ 18.2 MΩ.cm was obtained from a Sartorius Arium^®^ ultrapure water purifier system (Sartorius AG, Göttingen, Germany). Hermetic aluminium pans for DSC were purchased from DSC Consumables (Austin, MN, USA).

### 2.2. Characterisation

Characterisation of Polymers

Proton nuclear magnetic resonance (^1^H NMR) spectroscopy was performed on a 500 MHz Bruker Avance III HD spectrometer (Bruker BioSpin, Billerica, MA, USA). Gel permeation chromatography (GPC) was performed on a Shimadzu Prominence liquid chromatography system fitted with a differential refractive index detector (Shimadzu, RID-20A) and two Shimadzu columns (GPC-80MD and GPC-804D) connected in series (Shimadzu Corporation, Kyoto, Japan). THF was used as the eluent at a flow rate of 1 mL.min^−1^, and the columns were held at 40 °C. All samples were filtered through a 0.45 µm nylon syringe filter prior to injection. LabSolutions software (v5.93) (Shimadzu Corporation, Kyoto, Japan) was used to determine molecular weight characteristics with reference to a conventional column calibration.

Differential scanning calorimetry (DSC) of blends, PEG-b-PCL copolymers, and micelles was conducted on a Discovery Differential Scanning Calorimeter (TA Instruments, New Castle, DE, USA). The blends, copolymers, or copolymer micelles were weighed into aluminium pans using an Aczet Micro Balance CM 19 and sealed with a lid. For physical blends, PCL (*M_n_* = 2 kDa; 1 mg) and PEG (various molecular weights; 1 mg) powders were weighed into DSC pans and agitated using a vortex mixer. All experiments were conducted under a nitrogen flow (50 mL/min) to prevent oxidative degradation. The polymer samples were initially heated from −20 to 80 °C and then cooled to −65 °C with a ramp rate of 10 °C/min (first cycle). Subsequently, the samples were heated from −65 to 80 °C and then cooled to −65 °C a with ramp rate of 10 °C/min (second cycle). The average values and standard deviations of thermal transitions were calculated from replicate analysis (n = 5) of all samples (Supplementary Information (SI), Tables S1–S4). Selected DSC thermograms from individual sample analyses are provided in figures.

Wide-angle X-ray diffraction (WXRD) patterns were obtained using a Multi-Materials Analyser (MMA) X-ray Diffractometer (GBC Scientific Equipment, Braeside, VIC, Australia) with a Cu-Kα radiation source operating at a wavelength of λ = 1.541 Å, voltage of 30.0 kV, and a current of 30.0 mA. The polymer samples were scanned through the range of scattering angles (2θ) from 15 to 25° (*q* = 1.06–1.76 Å^−1^) at a rate of 1 °/min. Temperature-dependent diffraction patterns were recorded by placing the polymer sample on a temperature-controlled stage at 25 °C and recording the initial diffraction pattern. Subsequently, the samples were heated to 80 °C to record the molten state diffraction pattern and then cooled down at a rate of 10 °C/min to the temperature(s) of crystallisation (*T_cryst_*), and the diffraction pattern was recorded once again.

Synchrotron wide-angle X-ray scattering (WAXS) measurements were performed on the SAXS/WAXS beamline at the Australian Nuclear Science and Technology Organisation (ANSTO), Australian Synchrotron, Melbourne [28]. Samples were loaded into glass capillaries with a wall thickness of 0.01 mm and a nominal external diameter of 1.5 mm (Charles Supper, Natick, MA, USA) before being placed into a custom-built temperature-controlled capillary holder. The temperature of the capillary holder was controlled by a circulating water bath and set to 20 °C. The temperature during the WAXS experiments was monitored using a thermocouple, which was inserted into an empty capillary placed in the capillary holder. The detector configuration consisted of photon energy = 15.1 keV (wavelength, *λ* = 0.826 Å) with a sample-to detector distance of around 0.9 m. 2D scattering patterns were recorded for each sample using a Dectris Pilatus 2M detector, and the data were reduced to profiles of scattered X-ray intensity ((*I(q)*) versus the magnitude of the scattering vector *q* (= (4π/*λ*) sin*θ*, 2*θ* = scattering angle) using the in-house developed software Scatterbrain 2.82.

### 2.3. Procedures

Preparation of PCL homopolymer

Benzyl alcohol (0.208 mL, 2.00 mmol) was added to a round bottom flask (50 mL) complete with a stirrer bar under argon. ε-Caprolactone (20.0 mL, 175 mmol) and tin(II) 2-ethylhexanoate (0.648 mL, 2.00 mmol) were added sequentially, and the mixture was heated at 110 °C with stirring for 24 h. The mixture was cooled to ambient temperature to afford PCL_10_ as a white, waxy solid, 21.0 g (>98% isolated yield; >98.6% monomer conversion) (SI, Figure S1). ^1^H NMR (500 MHz, 25 °C, CDCl_3_) δ_H_ 1.32–1.38 (*m*, CH_2_, repeat unit (RU)), 1.58–1.65 (*m*, 2CH_2_, RU), 2.27 (*t*, CH_2_CO, RU), 3.61 (*t*, CH_2_OH, end-group), 4.02 (*t*, CH_2_O, RU), 5.08 (*s*, OCH_2_Ph, end-group), 7.27–7.34 (*m*, ArH, end group) ppm; *M_n_* = 10.1 kDa. GPC DRI (THF) versus PMMA standards: *M_w_* = 8.9 kDa, *Đ* = 1.50.

Preparation and lyophilisation of PEG-b-PCL copolymer micelles

Self-assembly of PEG-*b*-PCL copolymers in ultrapure water was conducted using a solvent evaporation approach. Polymer solutions were prepared in acetone (1 mL, 1 mg/mL) in vials (4 mL) and 1 mL of ultrapure water was then added dropwise. The vials were placed in a pre-heated block heater at 60 °C, and the solvent level was checked periodically until the volume was ~1 mL (~3 to 4 h). The vials were then placed in a vacuum desiccator (0.1 mbar) for 1 h to remove any residual organic solvent, and the solvent volume was readjusted with ultrapure water to ensure a polymer concentration of 1 mg/mL. The resulting micelle solutions were immediately frozen at −80 °C for 1 h and then lyophilised using an Alpha 1–2 LDplus (Martin Christ GmbH, Germany) freeze dryer for 72 h. The resulting lyophilised powders were stored in a desiccator at 21 ± 1 °C prior to analysis.

## 3. Results and Discussion

To interpret the relationship between the microstructure of PEG-*b*-PCL copolymers and their thermal characteristics when prepared as self-assembled micelles, it was imperative to first understand the behaviour of individual PEG and PCL homopolymers, their physical blends, and the copolymers prior to self-assembly. Therefore, we initially investigated the thermal characteristics of the homopolymers, blends, and copolymers to provide a comprehensive understanding of how the covalent tethering of the blocks influences their thermal transitions. Subsequently, this fundamental understanding was applied to interpret the thermal characteristics of self-assembled copolymer micelles.

### 3.1. Thermal Characterisation of PEG and PCL Homopolymers and Their Physical Blends

**Figure 1:**
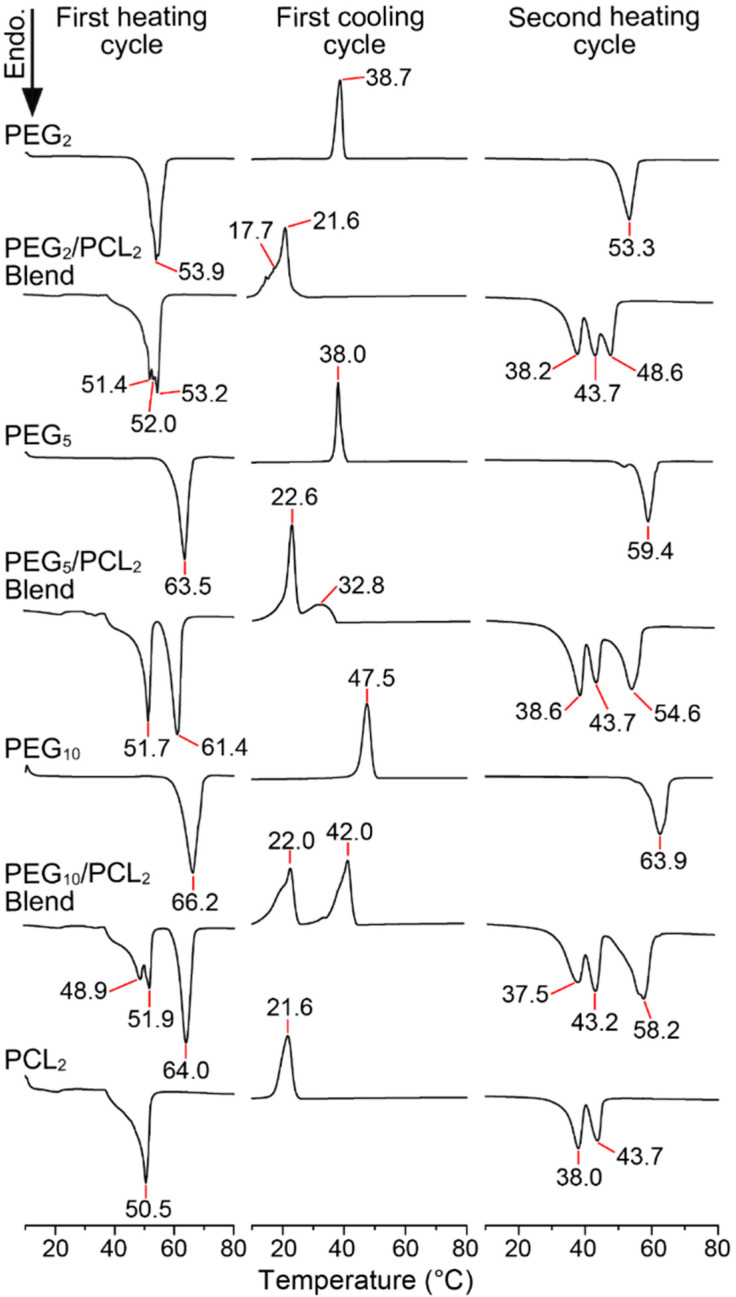
Representative DSC thermograms of PEG (*M_n_* = 2, 5 and 10 kDa) and PCL (*M_n_* = 2 kDa) homopolymers, and PEG/PCL homopolymer physical blends (1:1 *w*/*w*) showing the first heating, first cooling, and second heating profiles (ramp rate 10 °C /min).

The thermal history of a polymer is attributed to the crystalline characteristics (polymer morphology) of the polymer resulting from synthesis, processing, and storage conditions [29]. Therefore, the thermal behaviour of PEG (*M_n_* = 2, 5 and 10 kDa) and PCL (*M_n_* = 2 kDa) homopolymers and their physical blends were initially analysed via DSC through consecutive heating and cooling cycles (Figure 1).

For the PEG homopolymers, the melting temperatures (*T_m_*; melting peak maxima) and associated enthalpy of melting (∆H_m_) values were slightly reduced on the second heating cycle as compared to the first heating cycle (Figure 1 and SI, Table S1) following the elimination of the thermal history and were consistent with previously reported values [30]. The reduction in the ∆H_m_ values between heating cycles were associated with a decrease in the degree of crystallinity (*X_c_*) from 89–92% to 77–80%, where the ∆H_m(cryst)_ of 100% crystalline PEG was taken to be 205 J/g [31]. The slight increase in the *T_m_* and ∆H_m_ of the PEG homopolymers with increasing molecular weight is consistent with increasing degree of crystallinity [30,32,33].

While the *T_m_* of the PCL_2_ homopolymer on the first cycle was similar to the PEG_2_ homopolymer, the associated ∆H_m_ was significantly lower (SI, Table S1) and corresponded to a *X_c_* of only 56% (∆H_m(cryst)_ PCL = 140 J/g), although this does not account for differences in the strength of the intermolecular interactions responsible for crystallinity [34]. The first and second heating cycles for the PCL homopolymer revealed a unimodal to bimodal transition, which has been observed previously by others for low molecular weight PCL and attributed to the competing processes of crystallisation and phase separation that occur upon cooling [13,35]. Notably, only unimodal transitions were observed for higher molecular weight PCL, with the *T_m_* approaching 58–60 °C depending on the initial crystallisation temperature or cooling rate [35,36,37].

DSC thermograms of the PEG/PCL heterogeneous physical mixtures (1:1 *w*/*w*) revealed melting behaviour characteristics of both homopolymers in the first heating cycle, although it was evident that the initial melting of the PCL component (*T_m_* ~51 °C) resulted in a slight lowering of the PEG *T_m_* in the mixture as compared to the individual homopolymers (Figure 1). Upon cooling, all of the PEG/PCL blends displayed broad crystallisation peaks at *T_cryst_* ~22 °C consistent with the PCL homopolymer, indicating that the presence of PEG does not influence the *T_cryst_* of PCL. However, the *T_cryst_* of the PEG component in the blends was noticeably lowered in a molecular weight-dependent trend. For the PEG_2_/PCL_2_ and PEG_5_/PCL_2_ blends, the PEG *T_cryst_* was reduced to ~18 and ~33 °C, respectively, as compared to ~39 °C for the homopolymers. The reduction in PEG *T_cryst_* was also evident for the PEG_10_/PCL_2_ blend, and the complete separation of the crystallisation peaks for both the PEG and PCL components revealed that the ∆H_cryst_ was reduced by factors of ~4 and 1.5, respectively, as compared to their homopolymers (SI, Table S1). The reduced ∆H_cryst_ reflected a significant decrease in the *X_c_* of the PEG_10_ component of the blend from 80 to 17%, while a lower reduction from 46 to 31% was noted for the PCL_2_ component.

It is apparent that the crystallisation of PCL within the blends is weakly affected by the PEG component, whereas the PEG crystallisation is strongly affected by the PCL component. Furthermore, the initial crystallisation of the PEG component in the PEG_5_/PCL_2_ and PEG_10_/PCL_2_ blends would indicate that the two components are phase separated in the melt and that, upon cooling, the PEG component forms domains under confinement with reduced crystallinity within a continuous PCL phase [22,38]. This behaviour also explains the decrease in the *T_m_* and ∆H_m_ of the PEG component in the blends during the second heating cycle (Figure 1 and SI, Table S1), whereas the PCL component appears to be relatively unaffected compared to the PCL homopolymer alone. Taken together, these results indicate that, in the PEG/PCL blends, the thermal transitions of the individual components are distinguishable and that imperfect crystallisation of the PEG component occurs, which is consistent with previous observations [39].

### 3.2. Thermal Characterisation of PEG-b-PCL Copolymers

A family of PEG-*b*-PCL diblock copolymers was prepared via ring-opening polymerisation of caprolactone from PEG macroinitiators (Table 1) [27]. The covalent tethering of the blocks was anticipated to have a marked effect on their thermal behaviour as compared to physical blends. Therefore, DSC data from the first cooling and second heating cycles were analysed to investigate this parameter after the elimination of the thermal history caused by processing. The copolymers were purified via precipitation from chloroform (a good solvent for both blocks) into diethyl ether, which is a non-solvent for both blocks. The rapid transition from a solvated state to a solid state was hypothesised to limit the separation and crystallisation of the blocks, similarly to that observed when a homogeneous polymer melt is rapidly cooled [13]. Therefore, any *T_m_* transitions observed in the first heating cycle might be expected to be associated with a small ∆H_m_, as compared to the second heating cycle.

### 3.3. Thermal Characterisation of the PEG_2_PCL_y_ Copolymers Series

**Figure 2:**
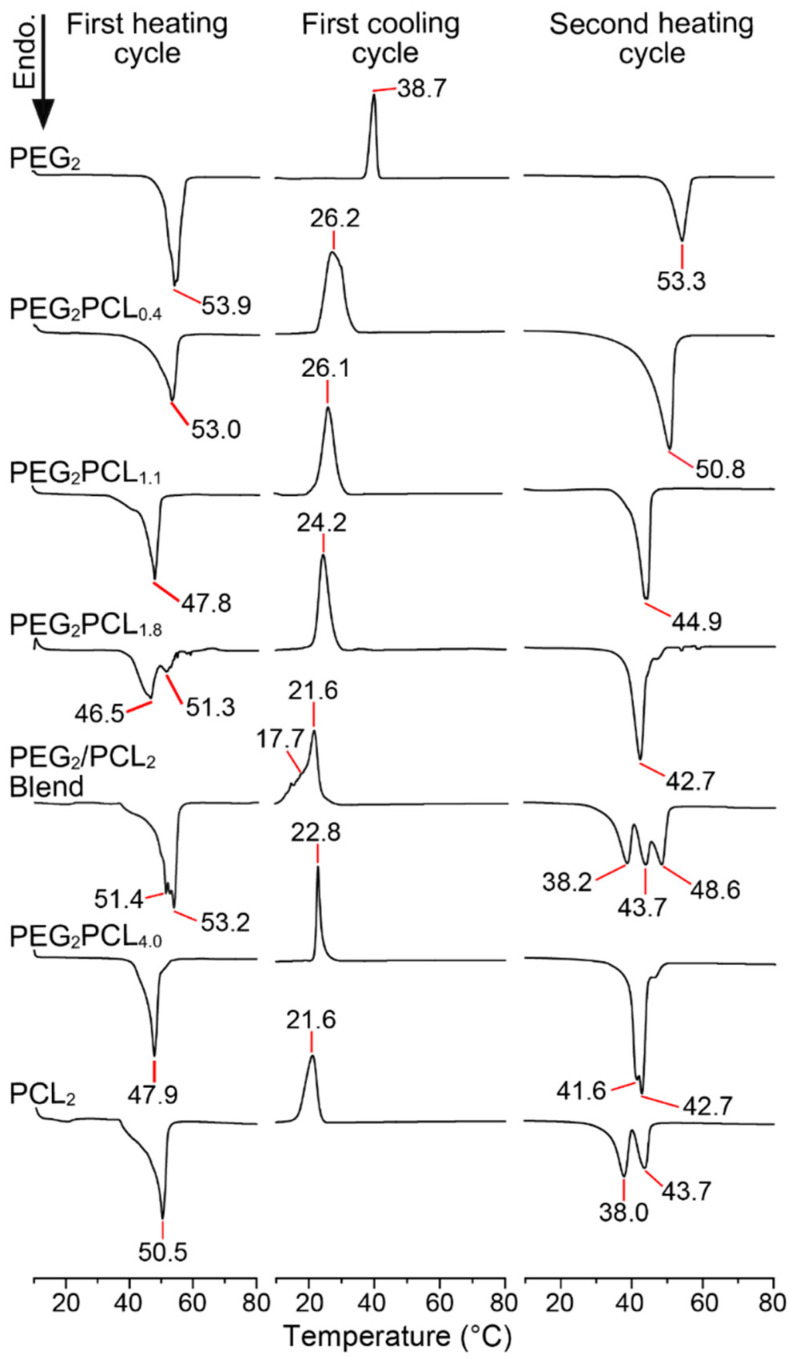
Representative DSC thermograms of the PEG_2_ and PCL_2_ homopolymers, PEG_2_PCL_y_ copolymer series, and PEG_2_/PCL_2_ (1:1 *w*/*w*) blend, showing the first heating, first cooling, and second heating profiles (ramp rate 10 °C/min).

DSC thermograms of the first heating cycle for the PEG_2_PCL_y_ copolymer series revealed the presence of distinct *T_m_* values, implying that crystallites were present despite the precipitation process, although it is not possible to confirm if the blocks are individually crystallised or co-crystallised (Figure 2). For the PEG_2_PCL_0.4_ copolymer, the *T_m_* was similar to that observed for the PEG_2_ homopolymer. Given the short PCL block, it is likely that only the PEG block crystallises. When the *f_PCL_* is increased, the *T_m_* is reduced to ~48 °C, and for the PEG_2_PCL_1.8_ copolymer a bimodal distribution was observed, possibly indicating the presence of separate PEG and PCL microdomains.

In the first cooling cycle, relatively narrow unimodal crystallisation peaks with similar *T_cryst_* values (23–26 °C) were observed for all the copolymers, which are significantly lower than the PEG_2_ homopolymer *T_cryst_* (~39 °C) and more consistent with the PCL_2_ homopolymer *T_cryst_* (~21 °C). Furthermore, the crystallisation peak shapes of the copolymers were distinctly different from that observed for the PEG_2_/PCL_2_ blend, indicating that the covalent tethering of the blocks influences the thermal behaviour of each block. However, from the DSC thermograms of the copolymers, it is not possible to determine which block has crystallised or if both have crystallised simultaneously.

**Figure 3:**
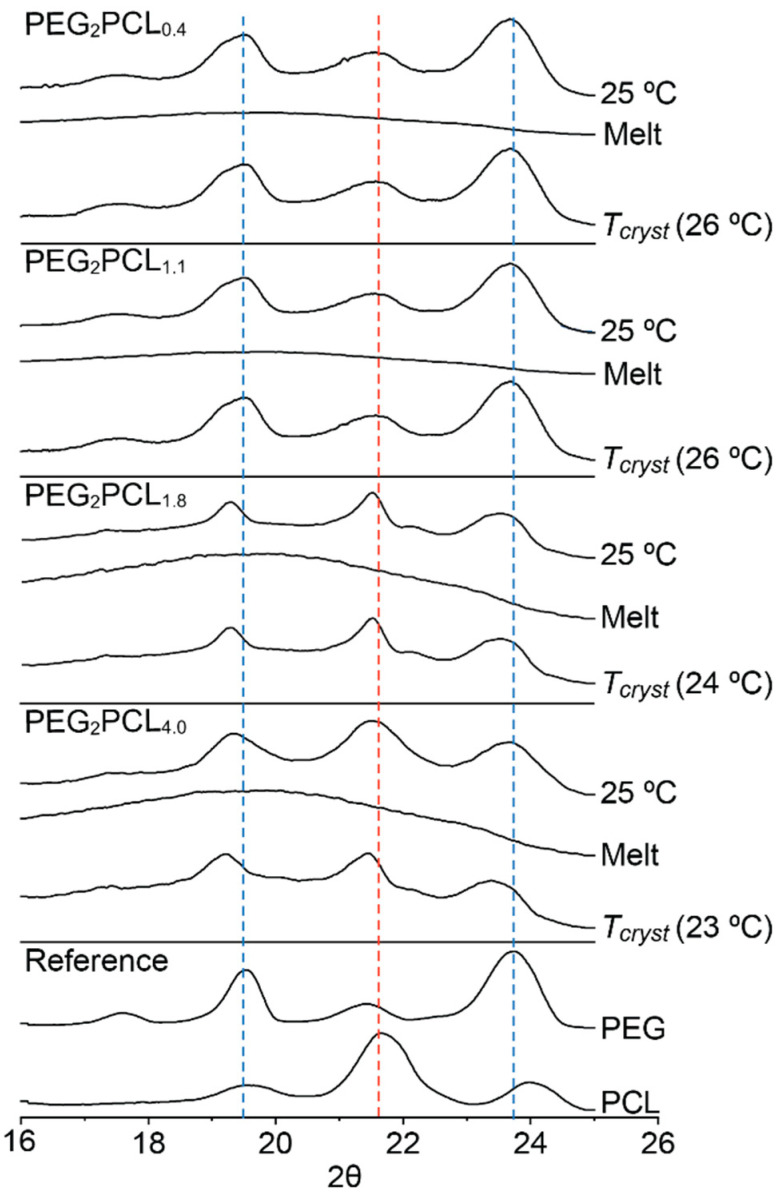
Stacked WXRD diffraction patterns of the PEG_2_ and PCL_2_ homopolymers recorded at 25 °C, and the PEG_2_PCL_y_ block copolymers recorded before thermal treatment at 25 °C (as precipitated), in the molten state at 80 °C, and at *T_cryst_* upon cooling.

Therefore, the crystallisation process was further investigated using temperature-dependent WXRD of the copolymers, which provided information about the extent of crystallisation and order of block crystallisation (Figure 3). WXRD patterns of the copolymers were recorded initially at 25 °C before thermal treatment (as precipitated), at 80 °C in the molten state, and at the *T_cryst_* upon cooling as determined from DSC (Figure 2). For reference, the WXRD patterns of the PEG_2_ and PCL_2_ homopolymers were initially recorded at 25 °C and revealed characteristic 2θ peaks at 19.2 and 23.7° for PEG and 21.8° for PCL [13,20,35]. Analysis of the copolymers before thermal treatment at 25 °C revealed the absence of the characteristic PCL peak for PEG_2_PCL_0.4_ and PEG_2_PCL_1.1_ copolymers, confirming that PCL is probably amorphous in these copolymers due to its short length and the influence of the larger PEG block. DSC thermograms of the copolymers cooled to −65 °C did not show any other crystallisation events (SI, Figure S2), confirming previous predictions that, when the PCL block is relatively small in length, it remains amorphous [39]. For the PEG_2_PCL_1.8_ and PEG_2_PCL_4.0_ copolymers, the diffraction pattern revealed characteristic peaks for both PEG and PCL (Figure 3), confirming that both blocks crystallise separately and simultaneously during precipitation and upon cooling of the melts.

**Figure 4:**
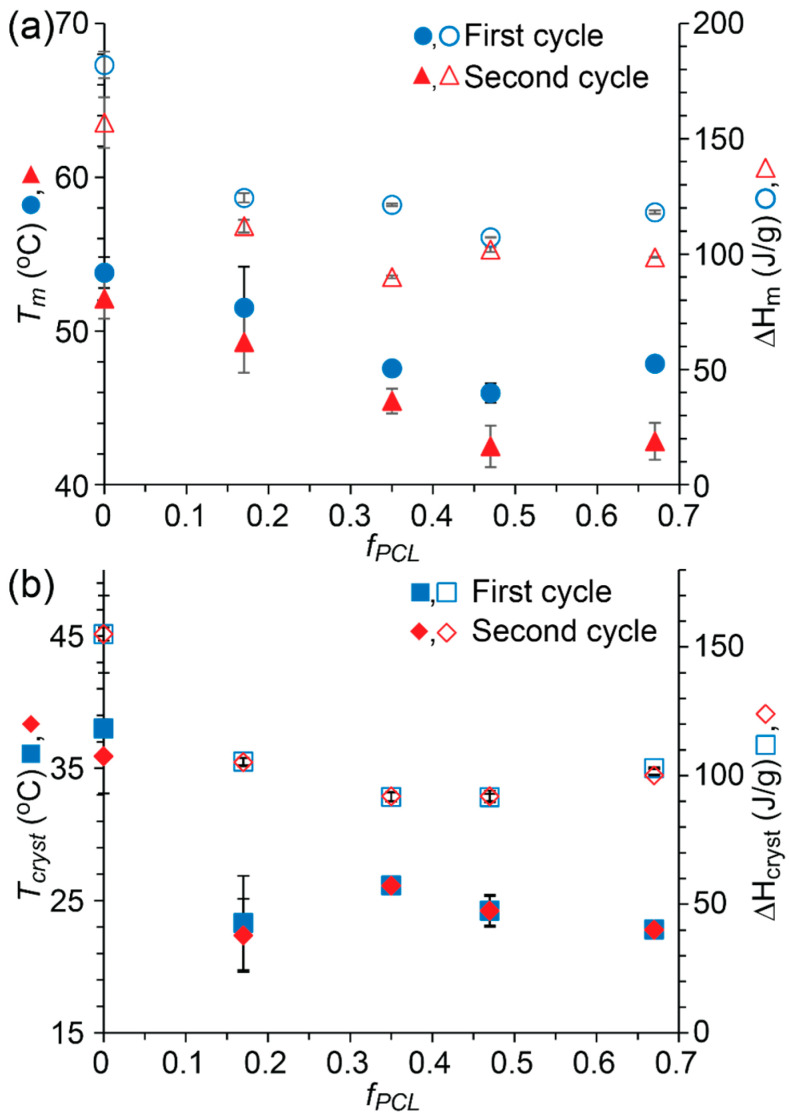
(**a**) *T_m_* and ΔH_m_, (**b**) *T_cryst_* and ΔH_cryst_ of the PEG_2_ homopolymer and PEG_2_PCL_y_ copolymer series recorded during the first and second heating cycles as a function of *f_PCL_* (n = 5).

Despite the apparent amorphousness of the PCL block for the PEG_2_PCL_0.4_ and PEG_2_PCL_1.1_ copolymers, the covalent tethering of the two blocks significantly depresses the *T_cryst_* and ∆H_cryst_ for the PEG block as compared to the PEG_2_ homopolymer (Figure 4 and SI, Table S2). This effect was also observed for the PEG_2_PCL_1.8_ and PEG_2_PCL_4.0_ copolymers but was accompanied by the simultaneous crystallisation of the PCL block. DSC thermograms of the copolymers recorded on the second heating cycle revealed a decrease in the *T_m_* with increasing *f_PCL_* (Figure 2), while the ∆H_m_ values remained consistent with those observed for the PEG_2_/PCL_2_ blend (Figure 4 and SI, Tables S1 and S2). Thus, for the PEG_2_PCL_0.4_ and PEG_2_PCL_1.1_ copolymers, even the presence of amorphous PCL causes a reduction in the PEG *T_m_*. For the PEG_2_PCL_1.8_ and PEG_2_PCL_4.0_ copolymers, the *T_m_* is reduced closer to that of the PCL_2_ homopolymer when the PCL block is also crystallised.

The depression of the PEG *T_m_* has been previously observed by Cerrai et al. for PCL-*b-*PEG-*b*-PCL triblock copolymers with a short PEG block (*M_n_ =* 950 Da) and different *f_PCL_*, whereby the crystallisation of the PCL block was found to occur first and to ‘freeze’ the polymer morphology, leading to imperfections in the PEG crystallites and depression of the PEG *T_m_* [40]. The depression of the *T_m_* of block copolymers results from a decrease in the lamellar thickness (*L*), which is strongly dependent on the interaction parameter between the two blocks as well as the volume fraction of each block [41]. For a block copolymer having an amorphous block and a crystalline block, imperfections in the crystallites are introduced when an amorphous layer is formed, which results in a decrease in *L*. Nojima et al. investigated the crystallisation behaviour of PCL-*b*-PEG-*b*-PCL triblock copolymers and found that increasing *f_PCL_* caused depression of the *T_cryst_* and *T_m_* due to a reduction in the *L* [39]. When the *f_PCL_* was very large (>0.85), WXRD revealed the absence of characteristic PEG diffraction peaks. It was therefore concluded that, during crystallisation, the minor component was rejected and accommodated in the space between the crystal lamellae in an amorphous state [25,39]. This behaviour explains the absence of PCL peaks in the WXRD patterns on the PEG_2_PCL_y_ copolymers with a *f_PCL_* < 0.35.

As observed in this study, an increase in the *f_PCL_* causes a decrease in the *T_m_*, which may result from a thinning of the PEG crystal lamellae (Figure 4). A reduction in the *L* would also explain the decrease in *T_cryst_* and ΔH_cryst_ in the PEG_2_PCL_y_ copolymer series when compared to the PEG_2_ homopolymer. In general, the *T_cryst_* of the copolymers remained between ~23 and 26 °C, regardless of the *f_PCL_*, which is slightly higher than the *T_cryst_* of the PCL homopolymer and significantly lower than the *T_cryst_* of the PEG homopolymer. He et al. found that PEG-*b-*PCL diblock copolymers possessed separate crystallisation peaks corresponding to PEG and PCL blocks, with PCL appearing to crystallise first when the *f_PCL_* > 0.60 followed by PEG in a confined microdomain [25]. In this study, the PEG_2_PCL_1.8_ (*f_PCL_* = 0.47) and PEG_2_PCL_4.0_ (*f_PCL_* = 0.67) copolymers did not show any separate crystallisation peaks even at higher *f_PCL_* (Figure 2), implying that the PCL and PEG blocks crystallise simultaneously without significant microphase separation, which results in a significant reduction in the *L* and decrease in *T_m_* and *T_cryst_*.

### 3.4. Thermal Characterisation of the PEG_5_PCL_y_ Copolymers Series

**Figure 5:**
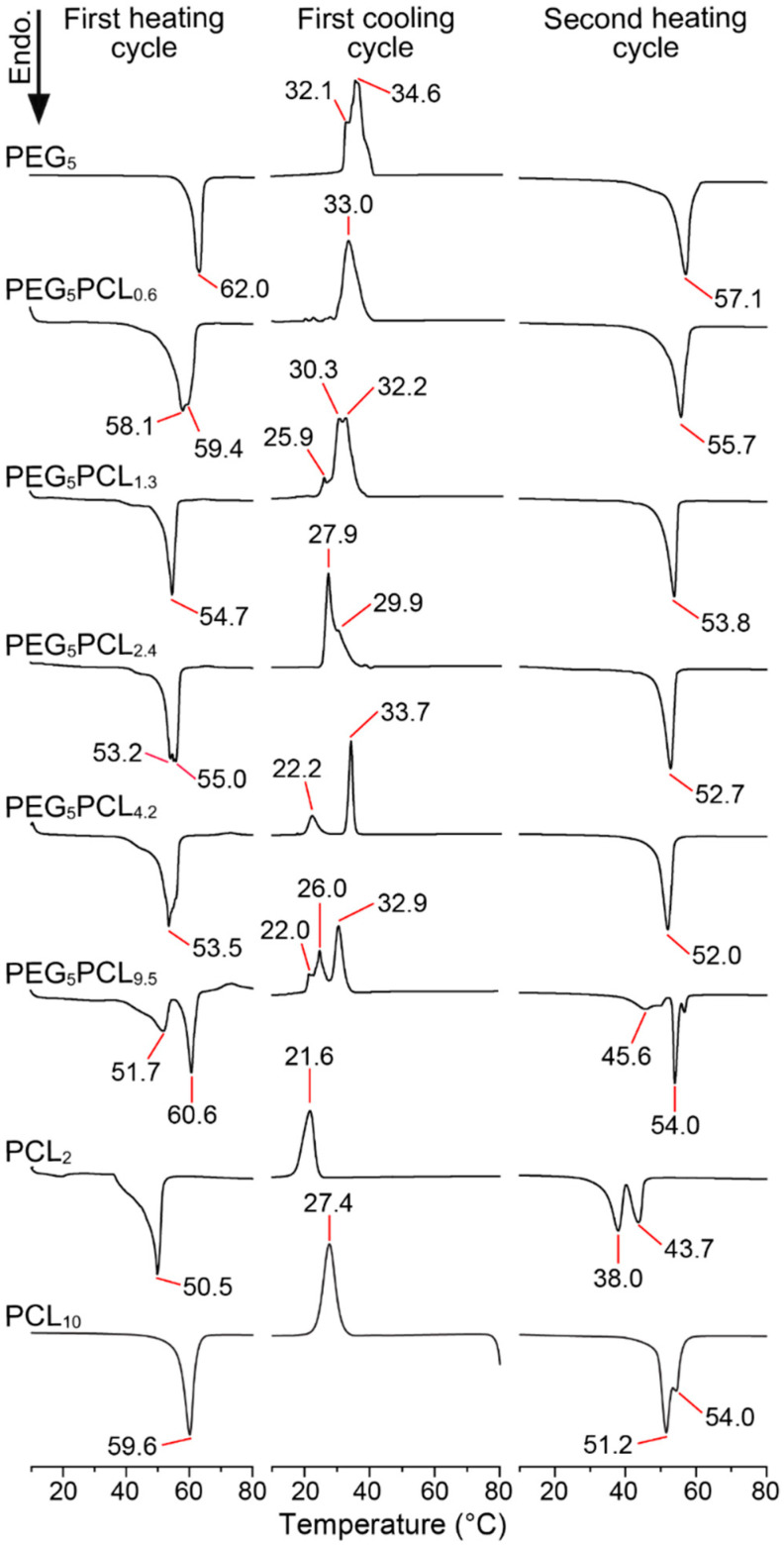
Representative DSC thermograms of the PEG_5_ and PCL homopolymers and the PEG_5_PCL_y_ polymer series, showing the first heating, first cooling, and second heating profiles (ramp rate 10 °C/min).

The thermal behaviour of the PEG_5_PCL_y_ copolymers series on the first and second heating cycles displayed similar trends to those observed for the PEG_2_PCL_y_ copolymer series, with the exception of the PEG_5_PCL_9.5_ copolymer that had the highest *f_PCL_* (Figure 5).

As compared to the PEG_5_ homopolymer on the first heating cycle (*T_m_* = 62 °C), the PEG_5_PCL_y_ copolymer series displayed slightly broader melting peaks with shoulders towards lower temperatures and reduced *T_m_* values (*T_m_* ~54–58 °C). On the second heating cycle, the copolymer melting peaks were sharper and the *T_m_* values reduced slightly with increasing *f_PCL_* (SI, Table S3 and Figure S3). For the PEG_5_PCL_9.5_ copolymer, two clear melting peaks were observed on the first heating cycle with *T_m_* values approximating the individual components, which indicated the presence of discrete crystalline microdomains. Similarly, two melting peaks were also observed on the second heating cycle, albeit with reduced *T_m_* and ΔH_m_ values. Similar results were also reported by Takeshita et al. for a PEG_5_PCL_10_ block copolymer, where it was determined that the *T_m_* values at lower and higher temperatures corresponded to PEG and PCL, respectively [42].

The first cooling cycle for the PEG_5_PCL_0.6_, PEG_5_PCL_1.3_ and PEG_5_PCL_2.4_ copolymers revealed broad peaks with *T_cryst_* and ΔH_cryst_ values significantly lower than those observed for the PEG_5_ homopolymer (SI, Figure 5 and Figure S3, Table S3), implying that the covalently tethered PCL block heavily influences the crystallisation of the PEG component, as observed for the PEG_2_PCL_y_ copolymer series. For the PEG_5_PCL_4.2_ and PEG_5_PCL_9.5_ copolymers, two distinct crystallisation peaks were observed.

**Figure 6:**
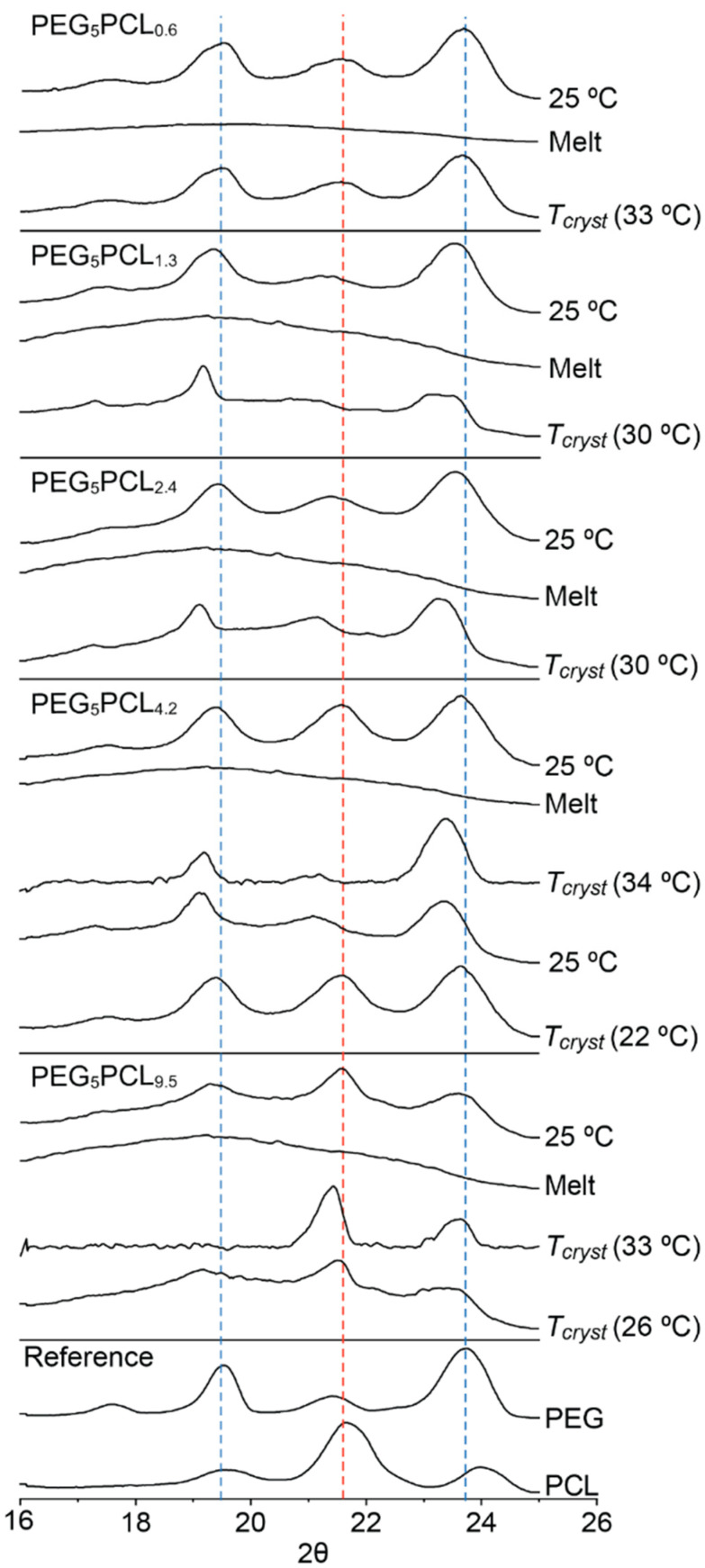
Stacked WXRD diffraction patterns of the PEG_5_ and PCL_2_ homopolymers recorded at 25 °C, and the PEG_5_PCL_y_ copolymer series at room temperature (25 °C), in the molten state (80 °C) and at the *T_cryst_* values upon cooling.

To determine which blocks were crystalline and the order of crystallisation, WXRD was conducted at various temperatures (Figure 6). Initially, the as-prepared PEG_5_PCL_y_ copolymer series was analysed at 25 °C, which revealed the presence of crystalline PEG in all copolymers. In comparison, the presence of a strong PCL peak was only noted for the PEG_5_PCL_4.2_ and PEG_5_PCL_9.5_ copolymers, suggesting that in copolymers with *f_PCL_* ≤ 0.32, the PCL block is predominately amorphous. Following removal of the thermal history, the copolymers were cooled to their *T_cryst_* and analysed again. For the PEG_5_PCL_0.6_, PEG_5_PCL_1.3_ and PEG_5_PCL_2.4_ copolymers, the diffraction patterns were similar to those recorded initially at 25 °C, with predominately crystalline PEG being present, although a contribution from crystalline PCL cannot be completely ruled out given that there is some overlap between the polymer peaks. Interestingly, the absence of a clear diffraction peak from the PCL component in the PEG_5_PCL_2.4_ copolymer (*f_PCL_* = 0.32) differs from that observed for the PEG_2_PCL_1.8_ copolymer (*f_PCL_* = 0.47) despite the shorter PCL block in the latter. This implies that it is primarily *f_PCL_* that controls PCL crystallisation in PEG-*b*-PCL copolymers and not the PCL molecular weight. This is further supported by the larger ΔH_cryst_ value for the PEG_5_PCL_2.4_ copolymer (99.4 ± 2.8 J/g) as compared to the PEG_2_PCL_1.8_ copolymer (91.6 ± 1.4 J/g), which can be predominately attributed to crystallisation of the PEG block.

While DSC thermograms revealed two distinct events upon cooling for the PEG_5_PCL_4.2_ and PEG_5_PCL_9.5_ copolymers, interestingly, WXRD revealed different orders of crystallisation of the two blocks in each case (Figure 6). For the PEG_5_PCL_4.2_ copolymer, the PEG component was found to crystallise prior to the PCL component. This behaviour is reversed for the PEG_5_PCL_9.5_ copolymer, with crystallisation appearing to be dominated by the PCL component, leading to subsequent imperfect PEG crystallisation, which correlates with the reduction in the *T_m_* of the PEG component observed in DSC (Figure 5). Nojima et al. similarly concluded from their work on PCL-*b*-PEG-*b*-PCL copolymers that at higher *f_PCL_*, PEG crystallisation is heavily influenced by PCL, resulting in a significant reduction in the *L* and the *T_m_* [39], as observed for the PEG_5_PCL_9.5_ copolymer. These results indicate that at a certain PEG molecular weight there is a threshold *f_PCL_* at which PCL begins to dominate the crystallisation process.

### 3.5. Thermal Characterisation of the PEG_10_PCL_y_ Copolymers Series

DSC thermograms of the PEG_10_PCL_y_ copolymer series on the first heating cycle revealed broad peaks with shoulders at lower temperatures and reduced *T_m_* values relative to the PEG_10_ homopolymer (SI, Figure S4). In comparison, the second heating cycle provided single narrow melting peaks for the copolymers (*T_m_* ~56 to 59 °C) despite clear evidence from the first cooling cycle that the PEG and PCL components crystallise separately for the PEG_10_PCL_7.9_, PEG_10_PCL_10.7_ and PEG_10_PCL_14.9_ copolymers. Furthermore, as the *f_PCL_* increased, there was an overall decrease in crystallinity, with the ΔH_cryst_ values for the PEG component decreasing (*X_c_* = 42 to 28%) with a concurrent increase in the ΔH_cryst_ values for the PCL component (*X_c_* = 16 to 20%) (SI, Table S4 and Figure S5). WXRD patterns of the PEG_10_PCL_3.2_ copolymer (*f_PCL_* < 0.24) revealed the absence of characteristic PCL diffraction peaks before or upon crystallisation, indicating that the PCL component remains amorphous (SI, Figure S6), as noted previously for copolymers with *f_PCL_* < 0.35. For the PEG_10_PCL_7.9_, PEG_10_PCL_10.7_, and PEG_10_PCL_14.9_ copolymers, the PEG component was found to crystallise first followed by crystallisation of the PCL component, indicating the formation of discrete crystalline microdomains.

### 3.6. Trends in the Thermal Behaviour of the PEG-b-PCL Copolymers

From a comparison of the results for all copolymers studied it can be concluded that the *f_PCL_* dominates the order and extent of crystallisation and that there are threshold *f_PCL_* values at which: (i) PEG crystallisation dominates and limits the crystallisation of PCL (*f_PCL_* < 0.35); (ii) PEG crystallises followed by PCL crystallisation (0.35 > *f_PCL_* < 0.65), and; (iii) PCL crystallises followed by PEG crystallisation (*f_PCL_* > 0.65). An exception to this is the PEG_2_PCL_4.0_ copolymer (*f_PCL_* of 0.67) in which PEG crystallises first, indicating that to a lesser extent, the PEG *M_n_* may also play a role at low overall polymer molecular weight. However, the PEG *M_n_* in combination with the *f_PCL_* does appear to be responsible for the segregation of crystalline microdomains. For all copolymers with *f_PCL_* < 0.35, the minor PCL component is likely accommodated in an amorphous layer between the PEG crystal lamellae. With the PEG_2_PCL copolymer series when *f_PCL_* > 0.35 the PEG and PCL components crystallise simultaneously without microphase separation. For the PEG_5_PCL and PEG_10_PCL copolymer series when *f_PCL_* > 0.35, there is a separation between the *T_cryst_* of the PEG and PCL components suggesting the formation of crystalline microdomains. In all cases, the covalent tethering of the two blocks results in a single melting peak on the second heating cycle with the exception of the PEG_5_PCL_9.5_ copolymer (Figure 5), indicating that, when crystallites of PEG and PCL are present, they melt at approximately the same temperature, which is not surprising given the similarity in the *T_m_* values for PEG and PCL homopolymers [32,33,35]. However, the observed *T_m_* values are typically lower than either that of the PEG or PCL homopolymers, which is consistent with imperfect crystallisation. For the PEG_5_PCL_9.5_ copolymer, further studies are required to identify which component melts first, but it could be argued that the initial crystallisation of PCL significantly reduces the crystallinity of the PEG component and, therefore, lowers its *T_m_* below that of PCL.

### 3.7. Characterisation of Lyophilised PEG-b-PCL Copolymer Micelles

Self-assembly of PEG-*b*-PCL copolymers in aqueous media results in the formation of micelles [27] in which the PEG and PCL components are inherently microphase segregated in the corona and core, respectively. During this self-assembly process, the PCL blocks aggregate together, which could result in their crystallisation, whereas the PEG block remains hydrated. However, upon lyophilisation the PEG corona could also crystallise, which would have implications for the rehydration and redispersion of the copolymer micelles. Conceptually, the lyophilised micelle powder is a solid dispersion, wherein PEG forms a continuous phase with PCL cores dispersed therein [4,14,21].

**Figure 7:**
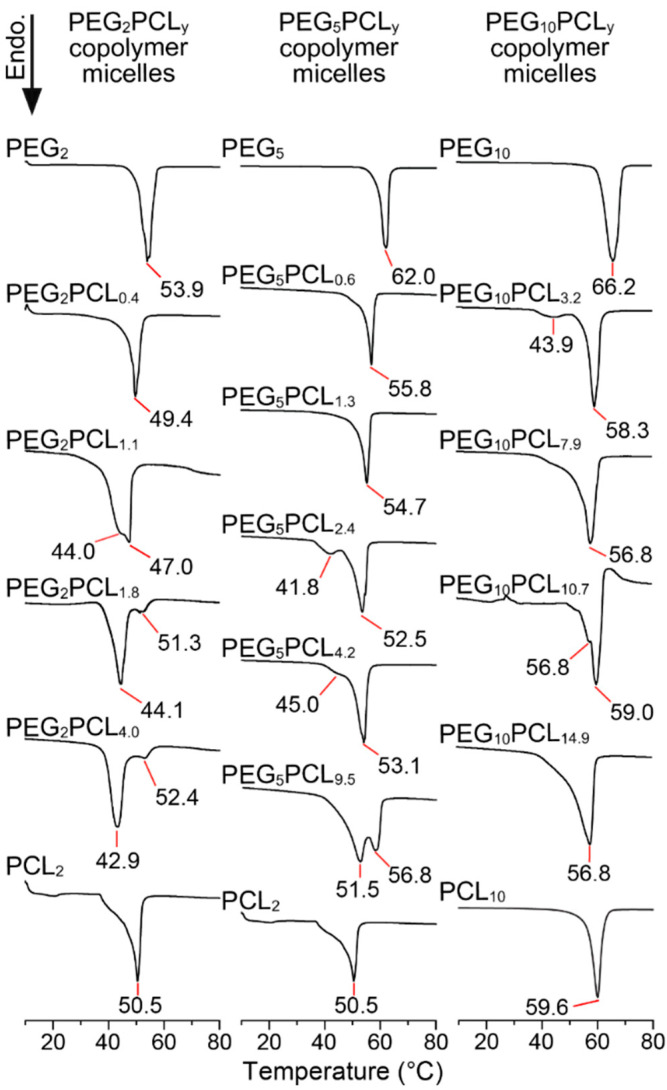
Representative DSC thermograms of the PEG and PCL homopolymers, and PEG_2_PCL_y_, PEG_5_PCL_y_, and PEG_10_PCL_y_ copolymer micelles (lyophilised powder), showing the first heating profiles (ramp rate 10 °C/min).

To assess the crystallinity of the core and corona of micelles formed from the PEG-*b*-PCL copolymers, lyophilised micelles prepared via the solvent evaporation technique were analysed by DSC, considering only the first heating cycle as melting would disrupt the as-prepared micellar structures (Figure 7). In both the PEG_2_PCL_y_ and PEG_5_PCL_y_ copolymer series, when *f_PCL_* ≤ 0.21, only single melting peaks with slight tails to lower temperatures and *T_m_* values close to the respective PEG homopolymers were observed in the thermograms. When *f_PCL_* ≥ 0.32, bimodal melting peaks were observed, although there were significant differences in the intensity of the peaks. For both copolymer series, *f_PCL_* values between 0.32 and 0.46 resulted in a dominant peak situated at higher temperatures with a lower intensity peak towards lower temperatures. Interestingly, this trend was reversed once *f_PCL_* ≥ 0.47, with the dominant peak at lower temperatures.

For both PEG_2_PCL_1.8_ and PEG_2_PCL_4.0_ micelles, the major *T_m_* peak occurs at ~43–44 °C, with a minor peak at ~52 °C. Interestingly, these are very similar to the peak locations observed for the PEG_5_PCL_2.4_ and PEG_5_PCL_4.2_ copolymer micelles with almost the same *f_PCL_*, except for a change in intensity (Figure 7). This could be an indication that there are fewer crystalline PCL domains, or a confinement effect of PCL being restricted to crystallise in the volume of the micelle core [43]. This is further supported by the fact that the PEG_10_PCL_3.2_ copolymer micelle also displays the same bimodal melting curve with peak intensities similar to PEG_5_PCL_2.4_ (Figure 7). Nevertheless, the appearance of melting peaks indicates the presence of polymer crystallites in the lyophilised micelle powder, which at first glance may be ascribed to the crystallisation of PCL in the micelle core during self-assembly. However, the presence of the double or bimodal melting peak indicates two different crystalline species (e.g., PEG and PCL crystallites) or different fold length/lamellae thicknesses [44,45]. The observed micelle trends differ significantly from the as-prepared copolymers in the bulk phase (Figure 2 and Figure 5 and SI, Figure S4) and imply that the self-assembly and lyophilisation processes result in the formation of unique crystalline microdomains. Unfortunately, WXRD of the micelles did not provide any evidence for PEG or PCL crystallisation, and therefore, the micelles powders were further analysed via synchrotron WAXS.

### 3.8. WAXS Analysis of Lyophilised PEG-b-PCL Copolymer Micelles

**Figure 8:**
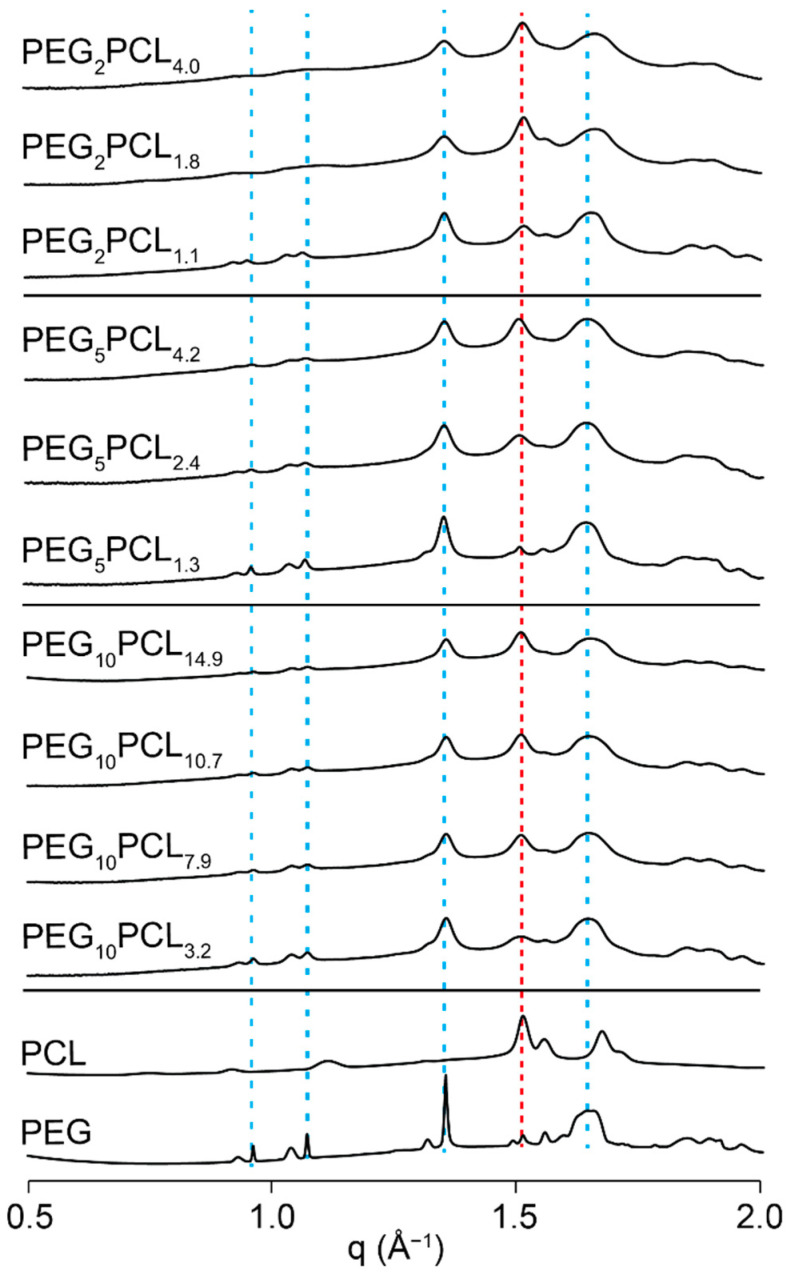
WAXS scattering profiles of the PEG_2_PCL_y_, PEG_5_PCL_y_, and PEG_10_PCL_y_ copolymer micelles (lyophilised powder), and PEG_5_ and PCL_2_ homopolymers.

From our previous studies, we have shown that the PEG_2_PCL_0.4_ and PEG_5_PCL_0.6_ copolymers do not form stable or well-defined micelles/aggregates, and therefore, they were omitted from WAXS analysis [27]. WAXS analysis of the PEG_5_ and PCL_2_ homopolymers revealed characteristic diffraction patterns over a wider scattering angle range than those recorded with WXRD (Figure 8) [46,47]. The PEG homopolymer displayed two major peaks at scattering vector (*q* = (4π/λ)sinθ, where 2θ is the scattering angle) values of ~1.35 and 1.65 Å^−1^, as well as several smaller sharp peaks around 0.9–1.1 Å^−1^. The PCL homopolymer displayed a major peak at ~1.50 Å^−1^ that overlapped slightly with a minor peak of PEG and was therefore used as an indicator of PCL crystallisation in the micelle cores. The diffraction patterns for the micelles indicated the presence of both PEG and PCL crystallites, with the intensity of the PCL peak showing a direct dependence on the *f_PCL_*. The fact that PEG crystallites are observed in all micelles implies that PEG crystallises during lyophilisation and/or upon storage. However, the broadening of the 1.35 Å^−1^ PEG peak (as well as others) in the micelles as compared to the homopolymer implies a decrease in the size of the crystallites and/or imperfections in the crystal lamellae [21,48].

For the PEG_2_PCL_1.1_, PEG_5_PCL_1.3_, PEG_5_PCL_2.4_, and PEG_10_PCL_3.2_ copolymer micelles (*f_PCL_* ~0.21 to 0.35), the PCL peak in the diffraction patterns is weak or absent (Figure 8), suggesting that the *f_PCL_* is insufficient to induce significant crystallisation, as was observed for the bulk copolymers, and that the micelle core is predominately amorphous. In addition, the presence of prominent and relatively well-defined PEG peaks at ~1 Å^−1^ for these micelles would imply that the amorphous core coincides with more ordered PEG crystallites in the corona. Another factor to consider is that the morphology of these micelles is cylindrical [27], and therefore, the PEG in the corona may adopt a more extended conformation (*cf.* spherical micelles) that promotes crystallisation. Additionally, the PEG grafting density has been reported to be higher at lower *f_PCL_*, which may favour crystallisation [49].

In the diffraction patterns of all other micelles with a *f_PCL_* ≥ 0.35, the PCL peak was clearly observable, although it varied in intensity relative to the PEG peaks. For example, the PEG_2_PCL_4.0_ micelles possessed a more intense PCL peak relative to the PEG peak (*cf.* PEG_5_PCL_4.2_ micelle), which may be indicative of a lower degree of PEG crystallisation with lower PEG *M_n_* or that the lower *M_n_* of PEG combined with higher *f_PCL_* allows for greater crystallisation of PCL core. The WAXS results also confirm that the melting peaks observed at lower and higher temperatures in the DSC thermograms (Figure 7) most likely correspond to the PCL and PEG components, respectively. In the case of the PEG_2_PCL_1.8_ and PEG_2_PCL_4.0_ micelles, high and low intensity peaks were observed at *T_m_* ~43 and 52 °C, respectively. The higher intensity peak observed for the PCL component is consistent with the higher intensity PCL peak in the diffraction patterns. The opposite trend is observed for the PEG_5_PCL_2.4_ and PEG_5_PCL_4.2_ micelles, with the smaller peak at *T_m_* ~42 °C in the thermogram corresponding to a reduction in the PCL peak intensity in the diffraction pattern.

For all copolymer series, the intensity of the PCL diffraction peak increased with increasing *f_PCL_*, indicating an increase in core crystallinity with increasing *f_PCL_*. Conversely, Glavas et al. reported that the crystallinity of the PCL core of PEG_2_-b-PCL_y_ micelles decreased (based on a decrease in ∆H_m_) with increasing *f_PCL_* from 0.33 to 0.66 [4,14], however, they conducted their thermal analysis on the copolymers in the bulk phase and not as lyophilised micelles. Indeed, our analysis of the PEG_2_-b-PCL_y_ micelles in bulk also revealed a decreasing trend in ∆H_m_ with increasing *f_PCL_* (Figure 4). Glover et al. performed DSC on as-prepared micelle solutions and found that the PCL core crystallinity of PEG_2_-b-PCL_y_ micelles decreased with increasing *f_PCL_* (~0.15 to 0.74) [21]. These results suggest that the lyophilisation process or storage of the PEG-*b*-PCL micelles in a lyophilised powder form results in crystallisation of the PCL core and that the self-assembly of the copolymers in water does not inherently result in core crystallisation nor is it driven by crystallisation. However, this does not account for the differences in core crystallisation at low *f_PCL_* observed in this study and that of Glover et al.

## 4. Conclusions

A comprehensive differential scanning calorimetry and wide-angle X-ray diffraction study of a library of PEG-*b*-PCL copolymers with PEG *M_n_* of 2, 5, and 10 kDa and *f_PCL_* ranging from 0.11 to 0.67 revealed that *f_PCL_* was the dominant factor responsible for controlling the thermal behaviour and morphology of the copolymers in the bulk phase. The *f_PCL_* dominated the order and extent of crystallisation of the two blocks, with *f_PCL_* < 0.35 leading to PEG crystallisation and amorphous PCL, 0.35 > *f_PCL_* < 0.65 resulting in PEG crystallisation followed by PCL crystallisation, and *f_PCL_* > 0.65 reversing this order of crystallisation. The PEG_2_PCL_4.0_ copolymer (*f_PCL_* of 0.67) was an exception to this trend, indicating that to a lesser extent, the PEG *M_n_* also plays a role at low molecular weights. Nevertheless, a combination of the PEG *M_n_* and *f_PCL_* appears to be responsible for the formation of crystalline microdomains. For all copolymers, when the *f_PCL_* < 0.35, the minor PCL component is most likely accommodated in an amorphous layer between PEG crystal lamellae. The absence of distinct crystallisation peaks for individual blocks of the PEG_2_PCL copolymer series when the *f_PCL_* > 0.35 implies that PEG and PCL crystallise simultaneously without microphase separation. In comparison, distinct crystallisation peaks for the two blocks of the PEG_5_PCL and PEG_10_PCL copolymer series when *f_PCL_* > 0.35 indicate the formation of crystalline microdomains for both blocks. A single melting peak is observed for all copolymers on the second heating cycle, with the exception of the PEG_5_PCL_9.5_, indicating that when PEG and PCL crystallites are present, they melt at the same temperature. The reduced enthalpy of crystallisation and melting of the copolymers compared to their homopolymers is consistent with imperfect crystallisation resulting from the covalent tethering of the two blocks or potential miscible regions where crystallisation is prevented. Synchrotron WAXS of lyophilised copolymer micelles revealed that PEG crystallisation occurs for all copolymers either during lyophilisation or upon storage. Similarly to the bulk copolymer powders, when *f_PCL_* < 0.35, PCL crystallisation in micelle cores was absent or very slight. In micelles, the extent of PCL crystallisation increased with increasing *f_PCL_*; however, it is likely that the lyophilisation process and storage conditions play roles in the core crystallisation for the micelles studied. These results provide insights into micelle core crystallisation that may prove valuable for interpreting drug encapsulation and release, particularly where micelle lyophilisation and reconstitution are employed.

## Figures and Tables

**Table 1:** PEG-*b*-PCL copolymer molecular weight characteristics, degree of polymerisation (DP), and weight fraction of hydrophobic block (*f_PCL_*).

Polymer Code	PEG *M_n_* (kDa) ^a^	PCL *M_n_* (kDa) ^a^	Copolymer *M_n_* (kDa) ^a^	*Ð* (*M_w_/M_n_*) ^b^	DP PEG; PCL	Weight Fraction of PCL (*f_PCL_*)
PEG_2_PCL_0.4_	2.0	0.4	2.4	1.03	45; 4	0.17
PEG_2_PCL_1.1_	2.0	1.1	3.1	1.02	45; 10	0.35
PEG_2_PCL_1.8_	2.0	1.8	3.8	1.02	45; 16	0.47
PEG_2_PCL_4.0_	2.0	4.0	6.0	1.03	45; 35	0.67
PEG_5_PCL_0.6_	5.0	0.6	5.6	1.01	114; 5	0.11
PEG_5_PCL_1.3_	5.0	1.3	6.3	1.02	114; 11	0.21
PEG_5_PCL_2.4_	5.0	2.4	7.4	1.02	114; 21	0.32
PEG_5_PCL_4.2_	5.0	4.2	9.2	1.03	114; 37	0.46
PEG_5_PCL_9.5_	5.0	9.5	14.5	1.09	114; 83	0.66
PEG_10_PCL_3.2_	10.0	3.2	13.2	1.02	227; 28	0.24
PEG_10_PCL_7.9_	10.0	7.9	17.9	1.04	227; 69	0.44
PEG_10_PCL_10.7_	10.0	10.7	20.7	1.04	227; 94	0.52
PEG_10_PCL_14.9_	10.0	14.9	24.9	1.12	227; 131	0.60

^a^ Number-average molecular weight (*M_n_*) calculated from ^1^H NMR spectroscopy. ^b^ Dispersity (*Ð*) calculated by GPC with reference to a conventional column calibration with narrow molecular weight PEG standards.

## Data Availability

The data presented in this study are available on request from the corresponding author.

## Supplementary Materials

The following supporting information can be downloaded at: https://www.mdpi.com/article/10.3390/polym14204365/s1, Figure S1: ^1^H NMR spectrum of PCL_10_ homopolymer; Figure S2: DSC thermograms of PEG_2_PCL_0.4_ and PEG_2_PCL_1.1_ copolymers; Figure S3: Graphed thermal transitions and enthalpy changes for the PEG_5_ homopolymer and PEG_5_PCL_y_ copolymer series; Figure S4: DSC thermograms of the PEG_10_ and PCL_10_ homopolymers and the PEG_10_PCL_y_ copolymer series; Figure S5: Graphed thermal transitions and enthalpy changes for the PEG_10_ homopolymer and PEG_10_PCL_y_ copolymer series; Figure S6: Stacked WXRD patterns of the PEG_10_PCL_y_ copolymer series; Table S1: Thermal properties of PEG and PCL homopolymers, and PEG/PCL physical blends; Table S2: Thermal properties of PEG_2_ and PCL_2_ homopolymers and PEG_2_PCL_y_ copolymer series; Table S3: Thermal properties of PEG_5_ and PCL homopolymers and PEG_5_PCL_y_ copolymer series; Table S4: Thermal properties of PEG_10_ and PCL_10_ homopolymers and PEG_10_PCL_y_ copolymer series.

## References

[1] Allen C., Maysinger D., Eisenberg A. (1999). Nano-engineering block copolymer aggregates for drug delivery. Colloids Surf. B Biointerfaces.

[2] Cabral H., Miyata K., Osada K., Kataoka K. (2018). Block copolymer micelles in nanomedicine applications. Chem. Rev..

[3] Gaucher G., Dufresne M.-H., Sant V.P., Kang N., Maysinger D., Leroux J.-C. (2005). Block copolymer micelles: Preparation, characterization and application in drug delivery. J. Control. Release.

[4] Glavas L., Odelius K., Albertsson A.-C. (2015). Tuning loading and release by modification of micelle core crystallinity and preparation. Polym. Adv. Technol..

[5] Sevgen E.S., de Pablo J.J., Hubbell J.A. (2018). A computational and experimental study of crystallization-driven self-assembly and micelle formation in poly (ethylene glycol)-b-oligo (ethylene sulfide). Biophys. J..

[6] Lu Y., Zhang E., Yang J., Cao Z. (2018). Strategies to improve micelle stability for drug delivery. Nano. Res..

[7] Petzetakis N., Dove A.P., O’Reilly R.K. (2011). Cylindrical micelles from the living crystallization-driven self-assembly of poly(lactide)-containing block copolymers. Chem. Sci..

[8] Kamaly N., Yameen B., Wu J., Farokhzad O.C. (2016). Degradable controlled-release polymers and polymeric nanoparticles: Mechanisms of controlling Drug release. Chem. Rev..

[9] Cheng F., Guan X., Cao H., Su T., Cao J., Chen Y., Cai M., He B., Gu Z., Luo X. (2015). Characteristic of core materials in polymeric micelles effect on their micellar properties studied by experimental and dpd simulation methods. Int. J. Pharm..

[10] Hua Z., Pitto-Barry A., Kang Y., Kirby N., Wilks T.R., O’Reilly R.K. (2016). Micellar nanoparticles with tuneable morphologies through interactions between nucleobase-containing synthetic polymers in aqueous solution. Polym. Chem..

[11] Letchford K., Burt H. (2007). A review of the formation and classification of amphiphilic block copolymer nanoparticulate structures: Micelles, nanospheres, nanocapsules and polymersomes. Eur. J. Pharm. Biopharm..

[12] Tanford C. (1974). Thermodynamics of micelle formation: Prediction of micelle size and size distribution. Proc. Natl. Acad. Sci. USA.

[13] An J.H., Kim H.S., Chung D.J., Lee D.S., Kim S. (2001). Thermal behaviour of poly (ε-caprolactone)-poly (ethylene glycol)-poly (ε-caprolactone) tri-block copolymers. J. Mater. Sci..

[14] Glavas L., Olsén P., Odelius K., Albertsson A.-C. (2013). Achieving micelle control through core crystallinity. Biomacromolecules.

[15] Alami-Milani M., Zakeri-Milani P., Valizadeh H., Salehi R., Jelvehgari M. (2018). Preparation and evaluation of PCL-PEG-PCL micelles as potential nanocarriers for ocular delivery of dexamethasone. Iran. J. Basic Med. Sci..

[16] Aliabadi H.M., Mahmud A., Sharifabadi A.D., Lavasanifar A. (2005). Micelles of methoxy poly (ethylene oxide)-b-poly (ɛ-caprolactone) as vehicles for the solubilization and controlled delivery of cyclosporine A. J. Control Release.

[17] Allen C., Han J., Yu Y., Maysinger D., Eisenberg A. (2000). Polycaprolactone–b-poly(ethylene oxide) copolymer micelles as a delivery vehicle for dihydrotestosterone. J. Control. Release.

[18] Gyun Shin I.L., Yeon Kim S., Moo Lee Y., Soo Cho C., Yong Kiel S. (1998). Methoxy poly(ethylene glycol)/ε-caprolactone amphiphilic block copolymeric micelle containing indomethacin. I. Preparation and characterization. J. Control Release.

[19] Latere D.J., Rouxhet L., Brewster M.E., Préat V., Ariën A. (2008). Spontaneously self-assembled micelles from poly(ethylene glycol)-b-poly(ε-caprolactone-*co*-trimethylene carbonate) for drug solubilization. Pharmazie.

[20] Mohanty A.K., Jana U., Manna P.K., Mohanta G.P. (2015). Synthesis and evaluation of MePEG-PCL diblock copolymers: Surface properties and controlled release behavior. Prog. Biomater..

[21] Glover A.L., Nikles S.M., Nikles J.A., Brasel C.S., Nikles D.E. (2012). Polymer micelles with crystalline cores for thermally triggered release. Langmuir.

[22] Luo C., Chen W., Gao Y. (2016). Fractional crystallization behavior of PCL and PEG in blends. Polym. Sci. Ser. A.

[23] Sun J., He C., Zhuang X., Jing X., Chen X. (2011). The crystallization behavior of poly(ethylene glycol)-poly(ε-caprolactone) diblock copolymers with asymmetric block compositions. J. Polym. Res..

[24] Bogdanov B., Vidts A., Van Den Buicke A., Verbeeck R., Schacht E. (1998). Synthesis and thermal properties of poly(ethylene glycol)-poly(ε-caprolactone) copolymers. Polymer.

[25] He C., Sun J., Deng C., Zhao T., Deng M., Chen X., Jing X. (2004). Study of the synthesis, crystallization, and morphology of poly(ethylene glycol)−poly(ε-caprolactone) diblock copolymers. Biomacromolecules.

[26] Ho R.-M., Chiang Y.-W., Lin C.-C., Huang B.-H. (2005). Crystallization and melting behavior of poly(ε-caprolactone) under physical confinement. Macromolecules.

[27] Faisal K.S., Clulow A.L., Krasowska M., Gillam T.A., Miklavcic S.J., Williamson N.H., Blencowe A. (2022). Interrogating the relationship between the microstructure of amphiphilic poly(ethylene glycol-b-caprolactone) copolymers and their colloidal assemblies using non-interfering techniques. J. Colloid Interface Sci..

[28] Kirby N.M., Mudie S.T., Hawley A.M., Cookson D.J., Mertens H.D.T., Cowieson N., Samardzic-Boban V. (2013). A low-background-intensity focusing small-angle X-ray scattering undulator beamline. J. Appl. Crystallogr..

[29] Eldridge J.E. (1967). Effect of the thermal history of polymers on their dynamic mechanical properties. J. Appl. Polym. Sci..

[30] Pielichowski K., Flejtuch K. (2003). Differential scanning calorimetry studies on poly (ethylene glycol) with different molecular weights for thermal energy storage materials. Polym. Adv. Technol..

[31] Martuscelli E., Silvestre C., Addonizio M.L., Amelino L. (1986). Phase structure and compatibility studies in poly(ethylene oxide)/poly (methyl methacrylate) blends. Makromol. Chem..

[32] Majumdar R., Alexander K.S., Riga A.T. (2010). Physical characterisation of polyethylene glycols by thermal analytical technique and the effect of humidity and molecular weight. Pharmasie.

[33] Sánchez-Soto P.J., Ginés J.M., Arias M.J., Novák C., Ruiz-Conde A. (2002). Effect of molecular mass on the melting temperature, enthalpy and entropy of hydroxy-terminated PEO. J. Therm. Anal. Calorim..

[34] Wu T., Shang X., Yang G. (2008). Comparison of crystallisation behaviors of poly (ε-caprolactone) in confined environment with that in bulk. J. Appl. Polym. Sci..

[35] Huang Y.P., Xu X., Luo X.L., Ma D.Z. (2002). Molecular weight dependence of the melting behavior of poly (ε -caprolactone). Chin. J. Polym. Sci..

[36] Abellah Ali A.F. (2016). Mechanical and thermal properties of promising polymer composites for food packaging applications. IOP Conf. Ser. Mater. Sci. Eng..

[37] Rusu M., Ursu M. (2006). Poly (vinyl chloride) and poly (e-caprolactone) blends for medical use. J. Thermoplast. Compos. Mater..

[38] Douglas P., Albadarin A.B., Sajjia M., Mangwandi C., Kuhs M., Collins M.N., Walker G.M. (2016). Effect of polyethylene glycol on the mechanical and thermal properties of bioactive poly (ε-caprolactone) melt extrudates for pharmaceutical applications. Int. J. Pharm..

[39] Nojima S., Ono M., Ashida T. (1992). Crystallisation of block copolymers II. Morphological study of poly (ethylene glycol)-poly (ε-caprolactone) block copolymers. Polym. J..

[40] Cerrai P., Tricoli M., Andrussi F., Paci M., Paci M. (1989). Polyether-polyester block copolymers by non-catalysed polymerisation of ɛ-caprolactone with poly (ethylene glycol). Polymer.

[41] Lauritsen J.I., Hoffman J.D. (1973). Extension of theory of growth of chain-folded polymer crystals to large undercoolings. J. Appl. Phy..

[42] Takeshita H., Fukumoto K., Ohnishi T., Ohkubo T., Miya M., Takenaka K., Shiomi T. (2006). Formation of lamellar structure by competition in crystallization of both components for crystalline–crystalline block copolymers. Polymer.

[43] Yu X., Wang N., Lv S. (2016). Crystal and multiple melting behaviors of PCL lamellae in ultrathin films. J. Cryst. Growth.

[44] Mihut A.M., Chiche A., Drechsler M., Schmals H., Di Cola E., Krausch G., Ballauff M. (2009). Crystallisation-induced switching of the morphology of poly (ethylene oxide)-block-polybutadiene micelles. Soft Matter.

[45] Reiter G. (2014). Some unique features of polymer crystallisation. Chem. Soc. Rev..

[46] Zhu Q., Harris M.T., Taylor L.S. (2011). Time-resolved SAXS/WAXS study of the phase behavior and microstructural evolution of drug/PEG solid dispersions. Mol. Pharm..

[47] Yang I.-K., Liu C.Y. (2010). Real-time SAXS and WAXS study of the multiple melting behavior of poly (ε-caprolactone). J. Polym. Sci. Part B Polym. Phys..

[48] Cheng S.S.D., Chen J., Barley J.S., Shang A., Habenschuss A., Sschack P.R. (1992). Isothermal thickening and thinning processes in low molecular-weight poly (ethylene oxide) fractions crystallised from the melt. 3. molecular weight dependence. Macromolecules.

[49] Shi B., Fang C., You M.X., Zhang Y., Fu S., Pei Y.Y. (2005). Stealth MePEG-PCL micelles: Effects of polymer composition on micelle physicochemical characteristics, in vitro drug release, in vivo pharmacokinetics in rats and biodistribution in S180 tumor bearing mice. Colloid Polym. Sci..

