# Supplementary material for: Microstructure—Thermal Property Relationships of Poly (Ethylene Glycol-b-Caprolactone) Copolymers and Their Micelles
Source: Polymers (Basel). 2022 Oct 16;14(20):4365. doi: 10.3390/polym14204365 (PMC9607102; doi:10.3390/polym14204365)
Supplement: Supplementary file 1 [file polymers-14-04365-s001.zip › polymers-1973865-supplementary.pdf]

# Supplementary Information

## Microstructure–thermal property relationships of poly(ethylene glycol-*b*-caprolactone) copolymers and their micelles

Khandokar Sadique Faisal,[a] Andrew J. Clulow,[b,c] Stephanie V. MacWilliams,[d] Todd A. Gillam,[a,d] Ashlyn Austin,[a] Marta Krasowska,[d] Anton Blencowe[a,*]

[a] Applied Chemistry and Translational Biomaterials (ACTB) Group, Centre for Pharmaceutical Innovation (CPI), UniSA Clinical and Health Sciences, University of South Australia, Adelaide, South Australia 5000, Australia

[b] BioSAXS beamline, Australian Synchrotron, ANSTO, 800 Blackburn Road, Clayton, Victoria 3168, Australia

[c] Drug Delivery, Disposition & Dynamics, Monash Institute of Pharmaceutical Sciences, 381 Royal Parade, Parkville, Victoria 3052, Australia

[d] Surface Interactions and Soft Matter (SISM) Group, Future Industries Institute, UniSA STEM, University of South Australia, Mawson Lakes, South Australia 5095, Australia

* Corresponding author emails: anton.blencowe@unisa.edu.au

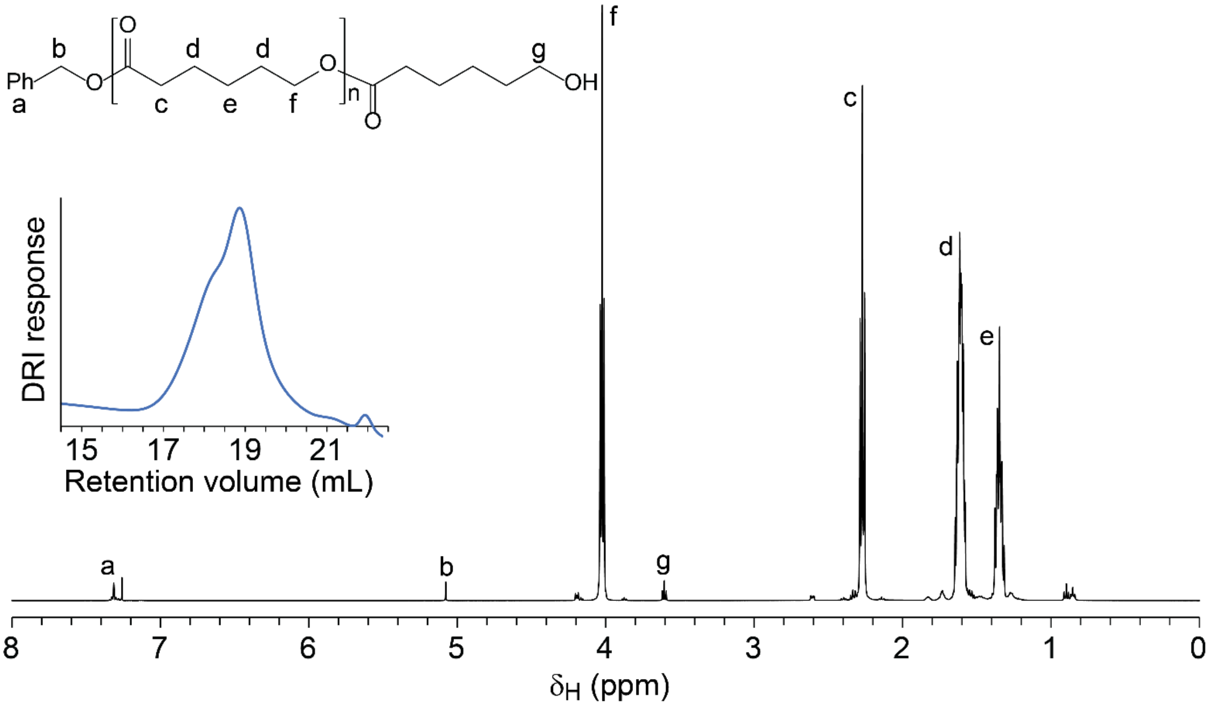


**Figure S1**: $^{1}H$ NMR spectrum (500 MHz, 25 °C, $CDCl_3$) of $PCL_{10}$ homopolymer. Inset: Gel permeation chromatography differential refractive index chromatogram of $PCL_{10}$ homopolymer.

**Table S1**: Thermal properties of PEG and PCL homopolymers, and PEG/PCL physical blends 1:1 (w/w) determined *via* DSC (n = 5) from consecutive heating/cooling cycles. Thermal properties include the $T_m$ and $T_{cryst}$, and the associated enthalpy changes ($\Delta H_m$ and $\Delta H_{cryst}$, respectively). Where melting or crystallisation peaks of the two components overlap a single $\Delta H_m$ or $\Delta H_{cryst}$ is reported for the thermal transitions of both components combined.

**First Cycle**

| Polymer Code | Polymer | $T_m$ (°C) | $\Delta H_m$ (J/g) | $X_c$ (%) | $T_{cryst}$ (°C) | $\Delta H_{cryst}$ (J/g) |
|---|---|---|---|---|---|---|
| $PEG_2$ | PEG | 53.8 ± 0.1 | 182 ± 5.8 | 89 | 38.0 ± 0.4 | 155 ± 2.6 |
| $PEG_2/PCL_2$ blend | PEG/PCL | 53.2 ± 0.2 | 108 ± 1.3 | | 21.6 ± 0.5 | 87.5 ± 1.3 |
| $PEG_5$ | PEG | 62.1 ± 1.5 | 189 ± 3.2 | 92 | 37.6 ± 0.4 | 157 ± 3.2 |
| $PEG_5/PCL_2$ blend | PEG | 61.4 ± 0.1 | 48.3 ± 0.1 | | 32.0 ± 0.5 | 81.9 ± 4.2 |
| | PCL | 51.7 ± 0.6 | 47.1 ± 0.8 | | 22.6 ± 0.9 | |
| $PEG_{10}$ | PEG | 66.2 ± 0.1 | 189 ± 3.2 | 92 | 45.8 ± 0.4 | 152 ± 10 |
| $PEG_{10}/PCL_2$ blend | PEG | 64.0 ± 0.9 | 52.7 ± 0.2 | | 42.0 ± 0.2 | 39.2 ± 0.6 |
| | PCL | 51.9 ± 0.6<br>49.7 ± 0.8 | 49.4 ± 0.6 | | 22.0 ± 0.9 | 45.7 ± 2.2 |
| $PCL_2$ | PCL | 50.8 ± 0.3 | 78.0 ± 4.1 | 56 | 21.9 ± 0.3 | 67.5 ± 0.7 |
| $PCL_{10}$ | PCL | 59.6 ± 0.1 | 114 ± 2.7 | 81 | 27.4 ± 0.1 | 85.2 ± 0.9 |

**Second Cycle**

| Polymer | | $T_m$ (°C) | $\Delta H_m$ (J/g) | $X_c$ (%) | $T_{cryst}$ (°C) | $\Delta H_{cryst}$ (J/g) |
|---|---|---|---|---|---|---|
| $PEG_2$ | PEG | 52.1 ± 1.3 | 157 ± 11 | 77 | 35.9 ± 2.8 | 155 ± 15 |
| $PEG_2/PCL_2$ blend | PEG | 48.6 ± 0.3 | 94.9 ± 2.4 | | 21.6 ± 0.7 | 87.4 ± 1.2 |
| | PCL | 43.7 ± 0.6<br>38.2 ± 0.1 | | | | |
| $PEG_5$ | PEG | 58.4 ± 1.1 | 158 ± 5.2 | 77 | 36.5 ± 1.7 | 152 ± 8.7 |
| $PEG_5/PCL_2$ blend | PEG | 55.1 ± 0.2 | 37.2 ± 0.8 | 18 | 32.8 ± 0.2 | 82.7 ± 3.5 |
| | PCL | 43.7 ± 0.7<br>38.7 ± 0.6 | 48.6 ± 1.3 | 36 | 22.4 ± 0.2 | |
| $PEG_{10}$ | | 62.5 ± 0.4 | 165 ± 4.5 | 80 | 45.9 ± 0.1 | 158 ± 6.2 |
| $PEG_{10}/PCL_2$ blend | PEG | 58.2 ± 0.4 | 36.5 ± 1.7 | 17 | 41.8 ± 0.4 | 39.3 ± 2.4 |
| | PCL | 45.2 ± 0.1<br>43.2 ± 0.3 | 43.5 ± 3.4 | 31 | 21.9 ± 0.1 | 42.0 ± 2.1 |
| $PCL_2$ | PCL | 38.1 ± 0.4<br>43.6 ± 0.3 | 64.7 ± 2.0 | 46 | 22.0 ± 0.3 | 66.1 ± 1.1 |
| $PCL_{10}$ | PCL | 51.2 ± 0.1<br>54.0 ± 0.1 | 84.5 ± 0.1 | 60 | 27.3 ± 0.1 | 85.2 ± 1.2 |

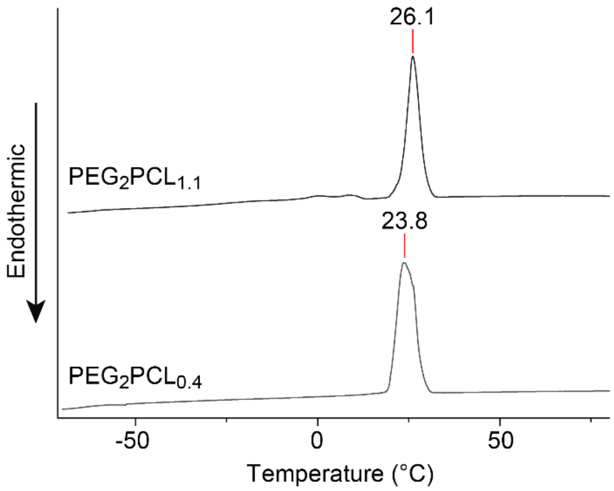


**Figure S2**: DSC thermograms of $PEG_2PCL_{0.4}$ and $PEG_2PCL_{1.1}$ copolymers showing the first cooling profile from 80 to -65 °C (ramp rate 10 °C/min).

**Table S2**: Thermal properties of $PEG_2$ and $PCL_2$ homopolymers and $PEG_2PCL_y$ copolymer series determined *via* DSC (n = 5) from the first and second heating/cooling cycles. Thermal properties include the $T_m$ and $T_{cryst}$, and the associated enthalpy changes ($\Delta H_m$ and $\Delta H_{cryst}$, respectively). Where melting or crystallisation peaks of the two components overlap a single $\Delta H_m$ or $\Delta H_{cryst}$ is reported for the thermal transitions of both components combined.

| | | | **First Cycle** | | |
|---|---|---|---|---|---|
| Polymer Code | Polymer | $T_m$ (°C) | $\Delta H_m$ (J/g) | $T_{cryst}$ (°C) | $\Delta H_{cryst}$ (J/g) |
| $PEG_2$ | PEG | 53.8 ± 1.0 | 182 ± 5.8 | 38.0 ± 0.4 | 155 ± 2.6 |
| $PEG_2PCL_{0.4}$ | PEG/PCL | 51.5 ± 2.7 | 124 ± 2.0 | 23.3 ± 3.6 | 105 ± 1.4 |
| $PEG_2PCL_{1.1}$ | PEG/PCL | 47.6 ± 0.2 | 121 ± 0.5 | 26.2 ± 0.6 | 91.7 ± 1.8 |
| $PEG_2PCL_{1.8}$ | PEG/PCL | 46.0 ± 0.6 | 107 ± 0.1 | 24.2 ± 1.2 | 91.6 ± 1.4 |
| $PEG_2PCL_{4.0}$ | PEG/PCL | 47.9 ± 0.2 | 118 ± 0.8 | 22.8 ± 0.2 | 103 ± 0.4 |
| $PCL_2$ | PCL | 50.8 ± 0.3 | 78.0 ± 4.1 | 21.9 ± 0.3 | 67.5 ± 0.7 |
| | | | **Second Cycle** | | |
| Polymer | | $T_m$ (°C) | $\Delta H_m$ (J/g) | $T_{cryst}$ (°C) | $\Delta H_{cryst}$ (J/g) |
| $PEG_2$ | PEG | 51.2 ± 1.3 | 157 ± 11 | 35.9 ± 2.8 | 155 ± 15 |
| $PEG_2PCL_{0.4}$ | PEG/PCL | 49.3 ± 2.0 | 112 ± 2.8 | 22.4 ± 2.8 | 105 ± 1.6 |
| $PEG_2PCL_{1.1}$ | PEG/PCL | 45.5 ± 0.8 | 90.1 ± 0.6 | 26.1 ± 0.6 | 91.9 ± 1.8 |
| $PEG_2PCL_{1.8}$ | PEG/PCL | 42.5 ± 1.4 | 102 ± 1.1 | 24.2 ± 1.1 | 91.9 ± 2.2 |
| $PEG_2PCL_{4.0}$ | PEG/PCL | 42.8 ± 1.2 | 98.6 ± 0.2 | 22.8 ± 0.2 | 100 ± 0.3 |
| $PCL_2$ | PCL | 38.1 ± 0.4<br>43.6 ± 0.3 | 64.7 ± 2.0 | 22.0 ± 0.3 | 66.1 ± 1.1 |

**Table S3**: Thermal properties of $PEG_5$ and PCL homopolymers and $PEG_5PCL_y$ copolymer series determined *via* DSC (n = 5) from the first and second heating/cooling cycles. Thermal properties include the $T_m$ and $T_{cryst}$, and the associated enthalpy changes ($\Delta H_m$ and $\Delta H_{cryst}$, respectively). Where melting or crystallisation peaks of the two components overlap a single $\Delta H_m$ or $\Delta H_{cryst}$ is reported for the thermal transitions of both components combined.

| | | | | | |
|---|---|---|---|---|---|
| | | **First Cycle** | | | |
| Polymer Code | Polymer | $T_m$ (°C) | $\Delta H_m$ (J/g) | $T_{cryst}$ (°C) | $\Delta H_{cryst}$ (J/g) |
| $PEG_5$ | PEG | 62.1 ± 1.5 | 189 ± 3.2 | 37.6 ± 0.4 | 157 ± 3.2 |
| $PEG_5PCL_{0.6}$ | PEG/PCL | 57.2 ± 0.5 | 120 ± 6.1 | 32.3 ± 0.4 | 104 ± 3.6 |
| $PEG_5PCL_{1.3}$ | PEG/PCL | 54.6 ± 0.1 | 125 ± 4.6 | 26.9 ± 1.6 | 116 ± 0.1 |
| $PEG_5PCL_{2.4}$ | PEG/PCL | 54.5 ± 0.6 | 109 ± 1.4 | 28.7 ± 0.7 | 99.4 ± 2.8 |
| $PEG_5PCL_{4.2}$ | PEG | 55.1 ± 1.6 | 119 ± 2.0 | 33.6 ± 0.1 | 107 ± 4.3 |
| | PCL | | | 22.0 ± 0.1 | |
| $PEG_5PCL_{9.5}$ | PEG | 60.2 ± 0.5 | 106 ± 7.1 | 26.3 ± 0.3 | 85.4 ± 7.0 |
| | PCL | 52.5 ± 0.8 | | 32.7 ± 0.1 | |
| $PCL_2$ | PCL | 50.8 ± 0.3 | 78.0 ± 4.1 | 21.9 ± 0.3 | 67.5 ± 0.7 |
| $PCL_{10}$ | PCL | 59.6 ± 0.1 | 114 ± 2.7 | 27.4 ± 0.1 | 85.2 ± 0.9 |
| | | **Second Cycle** | | | |
| Polymer | | $T_m$ (°C) | $\Delta H_m$ (J/g) | $T_{cryst}$ (°C) | $\Delta H_{cryst}$ (J/g) |
| $PEG_5$ | PEG | 58.4 ± 1.1 | 158 ± 5.2 | 36.5 ± 1.7 | 152 ± 8.7 |
| $PEG_5PCL_{0.6}$ | PEG/PCL | 55.7 ± 0.2 | 102 ± 7.5 | 36.3 ± 3.4 | 99.3 ± 12 |
| $PEG_5PCL_{1.3}$ | PEG/PCL | 53.9 ± 0.1 | 107 ± 3.4 | 31.4 ± 1.2 | 97.8 ± 1.8 |
| $PEG_5PCL_{2.4}$ | PEG/PCL | 52.8 ± 0.1 | 107 ± 6.0 | 28.0 ± 0.1 | 97.8 ± 0.2 |
| $PEG_5PCL_{4.2}$ | PEG | 52.1 ± 0.1 | 111 ± 5.2 | 33.6 ± 0.1 | 107 ± 3.9 |
| | PCL | | | 22.1 ± 0.1 | |
| $PEG_5PCL_{9.5}$ | PEG | 53.9 ± 0.1[a] | 79.3 ± 4.6 | 25.8 ± 0.7 | 87.1 ± 5.3 |
| | PCL | 45.6 ± 0.2[a] | | 36.2 ± 3.5 | |
| $PCL_2$ | PCL | 38.1 ± 0.4<br>43.6 ± 0.3 | 64.7 ± 2.0 | 22.0 ± 0.3 | 66.1 ± 1.1 |
| $PCL_{10}$ | PCL | 51.2 ± 0.1<br>54.0 ± 0.1 | 84.5 ± 0.1 | 27.3 ± 0.1 | 85.2 ± 1.2 |

[a] Assignment of the $T_m$ values for the individual components was not conclusively determined.

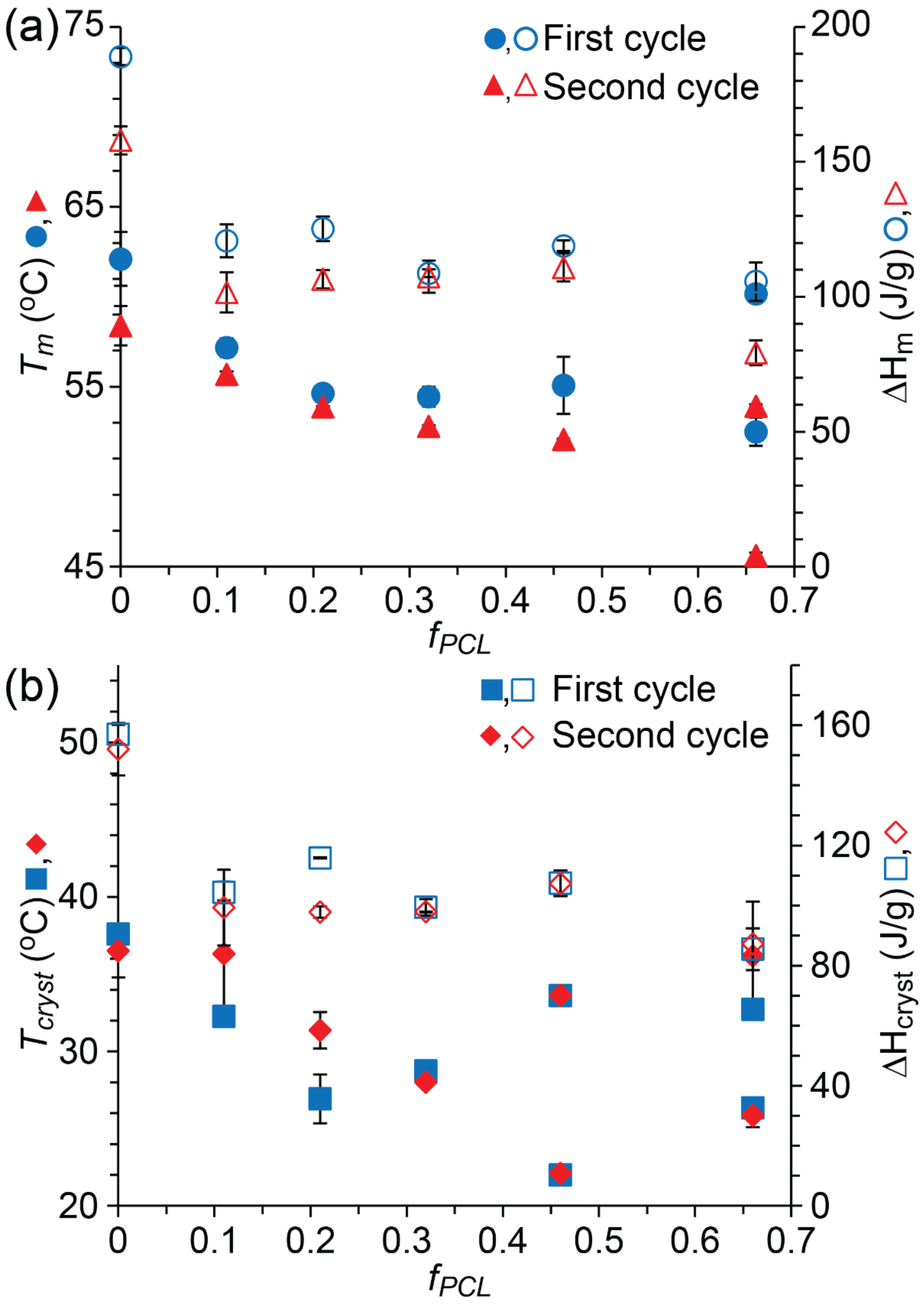


**Figure S3**: (a) $T_m$ and $\Delta H_m$, (b) $T_{cryst}$ and $\Delta H_{cryst}$ of the $PEG_5$ homopolymer and $PEG_5PCL_y$ copolymer series recorded during the first and second heating cycle as a function of $f_{PCL}$ (n = 5). For copolymers that display two distinct peaks in the DSC thermograms, individual values corresponding to each peak are shown.

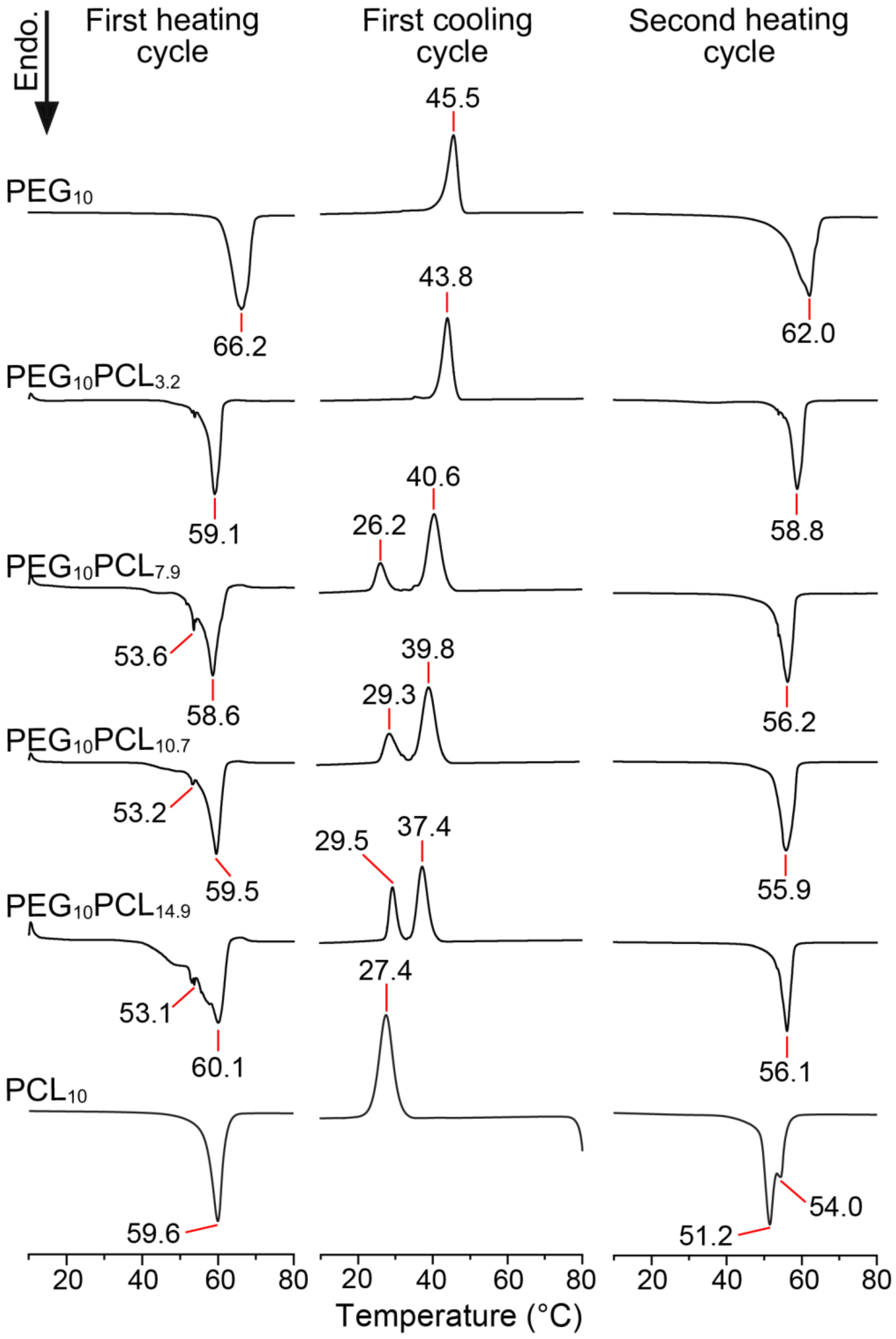


**Figure S4:** DSC thermograms of the $PEG_{10}$ and $PCL_{10}$ homopolymers and the $PEG_{10}PCL_y$ copolymer series showing the first heating, first cooling and second heating profiles (ramp rate 10 °C/min).

**Table S4**: Thermal properties of $PEG_{10}$ and $PCL_{10}$ homopolymers and $PEG_{10}PCL_y$ copolymer series determined *via* DSC (n = 5) from the first and second heating/cooling cycles. Thermal properties include the $T_m$ and $T_{cryst}$, and the associated enthalpy changes ($\Delta H_m$ and $\Delta H_{cryst}$, respectively). Where melting or crystallisation peaks of the two components overlap a single $\Delta H_m$ or $\Delta H_{cryst}$ is reported for the thermal transitions of both components combined.

| **First Cycle** | | | | | |
|---|---|---|---|---|---|
| Polymer Code | Polymer | $T_m$ (°C) | $\Delta H_m$ (J/g) | $T_{cryst}$ (°C) | $\Delta H_{cryst}$ (J/g) |
| $PEG_{10}$ | PEG | 66.2 ± 0.9 | 189 ± 2.3 | 46.2 ± 0.2 | 164 ± 2.7 |
| $PEG_{10}PCL_{3.2}$ | PEG/PCL | 58.9 ± 0.1 | 119 ± 3.8 | 43.7 ± 0.1 | 112 ± 3.3 |
| $PEG_{10}PCL_{7.9}$ | PEG | 57.0 ± 0.8 | 122 ± 1.0 | 40.4 ± 0.1 | 84.9 ± 0.3 |
| | PCL | | | 26.2 ± 0.1 | 22. 8 ± 3.1 |
| $PEG_{10}PCL_{10.7}$ | PEG | 59.2 ± 0.2 | 122 ± 1.1 | 39.6 ± 0.1 | 74.6 ± 5.9 |
| | PCL | | | 29.3 ± 0.8 | 24.8 ± 4.0 |
| $PEG_{10}PCL_{14.9}$ | PEG | 59.6 ± 0.3 | 107 ± 0.3 | 37.4 ± 0.1 | 56.9 ± 0.4 |
| | PCL | | | 29.4 ± 0.9 | 27.5 ± 0.4 |
| $PCL_{10}$ | PCL | 59.6 ± 0.1 | 114 ± 2.7 | 27.4 ± 0.1 | 85.2 ± 0.9 |
| **Second Cycle** | | | | | |
| Polymer | | $T_m$ (°C) | $\Delta H_m$ (J/g) | $T_{cryst}$ (°C) | $\Delta H_{cryst}$ (J/g) |
| $PEG_{10}$ | PEG | 62.1 ± 0.2 | 170 ± 1.4 | 46.4 ± 2.8 | 163 ± 4.2 |
| $PEG_{10}PCL_{3.2}$ | PEG/PCL | 58.6 ± 0.1 | 118 ± 3.0 | 43.8 ± 0.1 | 119 ± 0.3 |
| $PEG_{10}PCL_{7.9}$ | PEG | 56.2 ± 0.1 | 98.4 ± 4.7 | 40.7 ± 0.1 | 85.7 ± 0.5 |
| | PCL | | | 26.1 ± 0.1 | 22.2 ± 3.4 |
| $PEG_{10}PCL_{10.7}$ | PEG | 55.7 ± 0.1 | 108 ± 0.5 | 39.7 ± 0.1 | 68.3 ± 1.5 |
| | PCL | | | 29.2 ± 0.1 | 22.9 ± 4.8 |
| $PEG_{10}PCL_{14.9}$ | PEG | 56.1 ± 0.1 | 86.3 ± 2.4 | 37.3 ± 0.1 | 55.7 ± 1.3 |
| | PCL | | | 29.5 ± 0.1 | 28.5 ± 1.4 |
| $PCL_{10}$ | PCL | 51.2 ± 0.1<br>54.0 ± 0.1 | 84.5 ± 0.1 | 27.3 ± 0.1 | 85.2 ± 1.2 |

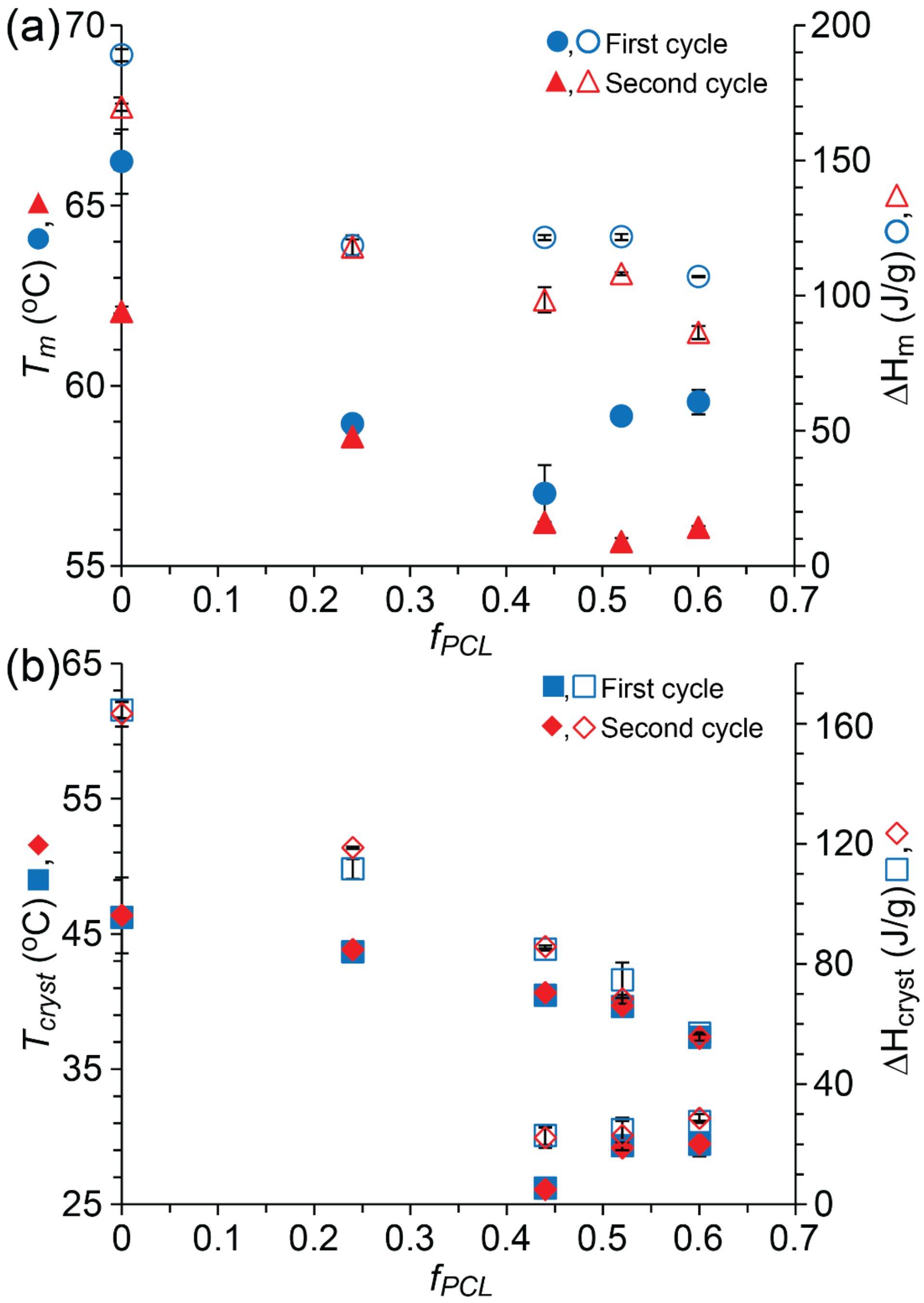


**Figure S5**: (a) $T_m$ and $\Delta H_m$, (b) $T_{cryst}$ and $\Delta H_{cryst}$ of the $PEG_{10}$ homopolymer and $PEG_{10}PCL_y$ copolymer series recorded during the first and second heating cycle as a function of $f_{PCL}$ (n = 5). For copolymers that display two distinct peaks in the DSC thermograms, individual values corresponding to each peak are shown.

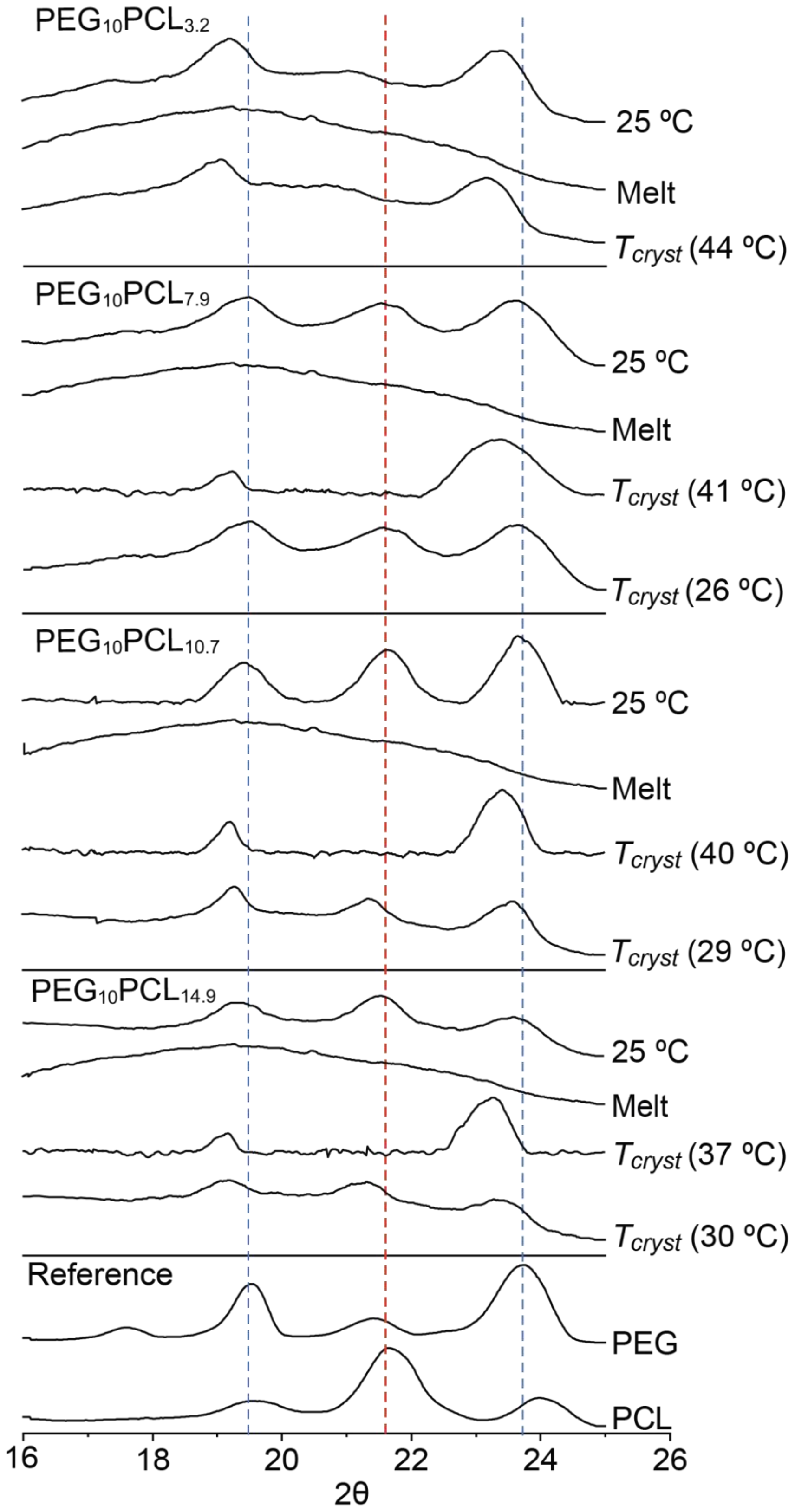


**Figure S6**: Stacked WXRD patterns of the $PEG_5$ and $PCL_2$ homopolymers recorded at 25 °C, and the $PEG_{10}PCL_y$ copolymer series at room temperature (25 °C), in the molten state (80 °C) and near the $T_{cryst}$ peak or between separate $T_{cryst}$ peaks.
